# Belatacept Use after Kidney Transplantation and Its Effects on Risk of Infection and COVID-19 Vaccine Response

**DOI:** 10.3390/jcm10215159

**Published:** 2021-11-03

**Authors:** Florian Terrec, Thomas Jouve, Paolo Malvezzi, Bénédicte Janbon, Hamza Naciri Bennani, Lionel Rostaing, Johan Noble

**Affiliations:** 1 Service de Néphrologie, Hémodialyse, Aphérèses et Transplantation Rénale, Centre Hospitalier Universitaire Grenoble Alpes (CHU), Université Grenoble Alpes, 38043 Grenoble, France; fterrec@chu-grenoble.fr (F.T.); tjouve@chu-grenoble.fr (T.J.); pmalvezzi@chu-grenoble.fr (P.M.); bjanbon@chu-grenoble.fr (B.J.); hnaciribennani@chu-grenoble.fr (H.N.B.); jnoble@chu-grenoble.fr (J.N.); 2 School of Medicine, Université Grenoble Alpes, 38043 Grenoble, France

**Keywords:** kidney transplantation, immunosuppressive regimen, opportunistic infections, belatacept, cytomegalovirus, CD80/CD86

## Abstract

Introduction: Belatacept is a common immunosuppressive therapy used after kidney transplantation (KT) to avoid calcineurin-inhibitor (CNI) use and its related toxicities. It is unclear whether its use exposes KT recipients (KTx) to a greater risk of infection or a poorer response to vaccines. Areas covered: We reviewed PubMed and the Cochrane database. We then summarized the mechanisms and impacts of belatacept use on the risk of infection, particularly opportunistic, in two settings, i.e., de novo KTx and conversion from CNIs. We also focused on COVID-19 infection risk and response to SARS-CoV-2 vaccination in patients whose maintenance immunosuppression relies on belatacept. Expert opinion: When belatacept is used de novo, or after drug conversion the safety profile regarding the risk of infection remains good. However, there is an increased risk of opportunistic infections, mainly CMV disease and Pneumocystis pneumonia, particularly in those with a low eGFR, in older people, in those receiving steroid-based therapy, or those that have an early conversion from CNI to belatacept (i.e., <six months post-transplantation). Thus, we recommend, if possible, delaying conversion from CNI to belatacept until at least six months post-transplantation. Optimal timing seems to be eight months post-transplantation. In addition, KTx receiving belatacept respond poorly to SARS-CoV-2 vaccination.

## 1. Introduction

For decades, calcineurin inhibitors (CNIs: cyclosporine A [CsA] or tacrolimus) have become the cornerstone of immunosuppressive therapy after kidney transplantation (KTx). However, these drugs are known to be nephrotoxic and can cause acute and/or chronic toxicity on the kidney graft [1,2], as well as contributing to new-onset diabetes [3] and hypertension [4]. Although the link between CNIs and chronic graft failure is not yet clear, it has led clinicians to seek new immunosuppressive regimens to avoid these drugs [5].

CNIs act on the first signal: thus, inhibition of the second signal is a promising approach. Thus, the more clinically relevant co-stimulation pathway that is targeted after organ transplantation, is the interaction between the clusters of differentiation (CD) CD28 and CD80 (B7-1)/86 (B7-2) [6,7]. The single agent that can interfere with this co-stimulation pathway and is currently used in clinical transplantation is belatacept that targets CD80/CD86 antigens.

Since the approval of belatacept in 2011 for use in the setting of de novo KTx, this CD80/86—CD28 co-stimulation blocker has been shown to be a valuable treatment option for maintenance immunosuppression. In this setting, belatacept has been associated with improved glomerular-filtration rates (GFR) compared to calcineurin inhibitor-based treatments because of the absence of nephrotoxicity [8,9].

Additionally, belatacept avoids cardiovascular side effects (e.g., hypertension and dyslipidemia) caused by a CNI-based-regimen [8] and improves glycemic parameters in diabetic KTx [10]. Nevertheless, de novo belatacept treatment causes a higher rate of acute rejection and a higher risk of lymphoproliferative disorders, especially in those that are seronegative for the Epstein--Barr virus (EBV) [8,9]. Bertrand et al. recently reported an enhanced potential higher risk of severe opportunistic infections (OIs) in a cohort of KTx [11]. It is also unclear if/how belatacept impacts with vaccination against COVID-19.

In this review, we look at the risk of opportunistic infections, including bacterial, viral, and fungal infections, and also focus on the COVID-19 infection and vaccination in the setting of belatacept-based immunosuppression.

## 2. COVID-19 and Belatacept

### 2.1. Vaccine Responses to mRNA COVID-19 Vaccines

It is now well known that, in contrast to immunocompetent individuals, a poor immune response to severe acute respiratory syndrome coronavirus 2 (COVID-19) mRNA vaccines has been observed in KTx. Several studies have demonstrated between 30 and 54% seroconversion rates in patients treated with CNI-based immunosuppressive therapy [12,13,14,15,16]. In this part of the review, we focus on the response to the COVID-19 vaccination in belatacept-treated KTx.

In April 2021, Chavarot et al. demonstrated, in a cohort of 101 patients that received belatacept with no history of COVID-19 infection that, at 28 days after the first vaccine injection, only two of the 101 patients (2.0%) developed anti-spike antibodies with the Pfizer Biontech vaccine. In addition, of the 35/101 (34.7%) patients tested for anti-spike serology at 1 month after the second dose, only two (5.7%) developed anti-spike antibodies. As for the specific T-cell response for the COVID-19, this was observed in two of 40 patients (5.0%) on day 28 and in seven of 23 patients (30.4%) at 1 month after the second injection [17].

The same team reported that, even with the third dose of Pfizer Biontech vaccine, performed between 26 and 95 days after the second injection, the immune response to the vaccine remained poor. Indeed 66 patients received three doses, of which only four patients (6.1%) developed antibodies. None had developed antibodies after the first or second dose. Two were treated with mycophenolic acid and the other two with azathioprine [18].

In the same study, Chavarot et al. [18] reported that the immune response to SARS-CoV-2 vaccination was better for patients with a history of COVID-19 infection before vaccination. Five KTx received two vaccine injections, including two patients that received three injections. Median IgG titer was 76 (39–290) AU/mL at the time of the first injection. The second dose was performed 28 days after the first injection; the time between the second and third injection was 40 (36–69) days. In contrast to KTx, with no prior COVID-19 infection before vaccination, all patients developed a strong antibody response after vaccination [18].

It has to be noted that, in these two studies, belatacept infusion and the vaccine injections were performed on the same days. Moreover, all patients were treated with steroids in association with belatacept.

Recently Bertrand et al. published a study that compared the immune response to Pfizer Biontech given to KTx receiving tacrolimus-based immunosuppression (24 patients), belatacept-based immunosuppression (10 patients), and a third heterogeneous group receiving a non-tacrolimus or belatacept immunosuppressive regimen (11 patients, 72% treated with CsA). At one month after the second injection, two KTx (8.3%) had developed anti-SARS-CoV-2 antibodies in the tacrolimus-treated group, zero KTx developed anti-SARS-CoV-2 antibodies in the belatacept-treated group, and six KTx (54.5%) developed anti-SARS-CoV-2 antibodies in the third group. At one month after the second injection, 12 KTx (50%) in the tacrolimus-treated group, four KTx (40%) in the belatacept-treated group, and 10 KTx (90.9%) in the third group displayed a specific T-cell response.

This study confirms the poor response to the vaccine in patients treated with belatacept [19].

In August 2021, Noble et al. published a study examining the immune response at post-SARS-CoV-2 vaccination. Fifty-seven patients were recruited: mean age was 62 ± 13 years. Immunosuppression was belatacept for 41 patients (72%) and tacrolimus for 16 patients (28%). Overall, 21 patients (36%) had a positive immune response at post-SARS-CoV-2 vaccination (after the third dose). Of these, after the second dose, seven (17%) belatacept-treated patients and nine (56%) tacrolimus-treated patients tested positive for anti-SARS-CoV-2 antibodies (*p* = 0.003). After the third vaccination, 20 belatacept patients were assessed: four patients were positive (20%). In this study, all patients received a vaccination at 21 days after belatacept infusion. This may explain the improved response to vaccination (i.e., 17%) after the second vaccination. However, it has to be noted that none of these patients was receiving steroids [20].

#### 2.1.1. Expert Opinion

Belatacept significantly reduces the immune response to SARS-CoV-2 mRNA vaccination. The injection of the third dose of the vaccine also seems to be inefficient. However, this immune response is better for patients with a history of COVID-19 infection. In light of the high mortality of subjects receiving immunosuppression, those surviving the infection are likely to have a “better/fitter” immune system. In turn, this could explain their better response to vaccination. Delaying COVID-19 vaccination until 21 days after a belatacept infusion and steroid avoidance may improve immunogenicity.

Regarding the present COVID-19-related pandemic, we recommend conducting a complete vaccination regime before converting a patient to belatacept.

This poor humoral response could be explained by several underlying mechanisms: belatacept plays a direct and active role at several steps in the humoral response, reducing plasmablast differentiation, immunoglobulin production, and expression of major transcription factors involved in plasma-cell function [21]. Moreover, belatacept modulates B cell-Tfh crosstalk, leading to the impaired germinal-center formation and improper antibody response in KTx treated with belatacept [21,22]. Interestingly, Salter et al. have recently shown that most spike-specific Th cells express the co-activating molecule CD28; and could therefore become theoretically inhibited by belatacept [23].

Hence, while awaiting new strategies to improve the immunogenicity of anti-COVID-19 vaccines, such as the use of a higher antigen dose or a third booster dose, or to test the efficacy of other approved vaccines, belatacept-treated KTx should maintain enhanced barrier measures. Vaccination of household members could also confer indirect protection to KTx.

One alternative for non-responder patients is prophylaxis with Ronapreve. This treatment comprises two monoclonal antibodies, casirivimab and imdevimab, targeting the SARS-CoV-2 spike protein to reduce the risk and severity of COVID-19 infection in selected patients.

This treatment was tested as prophylaxis in a study by O’Brien et al. in August 2021. They evaluated the prophylactic subcutaneous administration of 1200 mg of casirivimab/imdevimab versus a placebo given to people without antibodies to COVID-19 (seronegative) and that had been exposed to SARS-CoV-2 from someone in their household. Those that initially had a negative PCR result were less likely to develop the disease when administered casirivimab/imdevimab within 96 h of contact (relative risk reduction 66%, *p* < 0.001); in addition, any consequent symptomatic infections also resolved faster (1.2 weeks vs. 3.2 weeks). In asymptomatic individuals with an initial positive PCR result, treatment with casirivimab/imdevimab reduced the development of symptomatic disease by 31% (*p* < 0.038) [24]. However, the cost and limited supply of casirivimab/imdevimab emphasizes the need to ensure it is allocated to those that stand to gain the most benefit: seronegative SARS-CoV-2 patients after three injections of the vaccine and receiving belatacept could be good candidates [25].

#### 2.1.2. COVID-19 Infection and Belatacept

Theoretically, solid organ transplanted (SOT) recipients are at an exceptionally high risk of developing critical COVID-19 due to chronic immunosuppression. Unfortunately, only a few data regarding the incidence of COVID-19 and its severity in patients receiving belatacept have been published.

In 2020, Marx et al. reported rapid recovery from COVID-19 in a patient receiving belatacept-based immunosuppression. This patient presented with no cytokine release syndrome either. The authors hypothesized that the mild clinical course of COVID-19 observed in their patients may have been, at least partially, due to a belatacept-related blockade of massive cytokine/chemokine production [26].

Kamar et al. reported recently on 460 SOT recipients; of these 23 were hospitalized for confirmed COVID-19. Among belatacept-treated patients, there was only one case of COVID-19 infection. A chest CT showed multiple patchy ground-glass opacities. The patient was hospitalized, belatacept was discontinued, and no cytokine storm was noted. He was discharged one month later [27].

In October 2020, Elias et al. studied 1216 patients that had undergone kidney transplantation and had been actively followed up in two transplantation centers in Paris. Of these, 66 (5%) patients were diagnosed with COVID-19 disease between 1 March and 30 April 2020. Six patients were receiving maintenance belatacept, and perfusion of belatacept was delayed for one patient. Six of 70 patients treated with belatacept (8.5%) had a COVID-19 infection vs. 5.3% in the tacrolimus-regimen group and 5.4% for the overall non-belatacept group. The six belatacept-treated patients were hospitalized: five with non-invasive mechanical ventilation and one with invasive mechanical ventilation. Two of them died (2.8%) vs. 1.4% in the tacrolimus group and 1.4% in the overall non-belatacept group. However, these differences were not statistically significant [28].

Chavarot et al. followed 181 belatacept-treated KTx: all received ≥ 2 COVID-19 BNT162b2 vaccines. Of these, 12 (6.6%) developed symptomatic COVID-19 at a median time of 18 (8–30) days after their last vaccine injection, including nine (5.0%) at ≥14 days after the second vaccination (breakthrough COVID-19). All 12 patients presented with more than two comorbidities. COVID-19 developed after the first injection in three patients, and after the second injection in eight patients, at a median time of 31.5 (15–58) days after injection. One patient also developed COVID-19 six days after the third dose. Eight (67%) patients required hospitalization, including three in an intensive care unit. Six (50%) patients died. Causes of death were an acute respiratory failure in four patients, myocardial infarction in one, and septic shock in one [18].

#### 2.1.3. Expert Opinion

When infected by SARS-CoV-2, there is a trend for more hospitalizations overall and cases in ICUs, and more deaths of belatacept-treated KTx compared to a CNI-based treatment. However, regarding the absence of solid data, including prospective, controlled trials, it is difficult to ascertain that this increased risk is related to the belatacept treatment.

Nevertheless, acute respiratory distress syndrome, caused by coronaviruses (SARS-CoV, MERS-CoV, SARS-CoV-2), is characterized by a cytokine storm, with the secretion of numerous proinflammatory cytokines [29,30]. The mild clinical course of COVID-19 observed in several case reports may be, in part, related to the belatacept-related blockade of massive cytokine/chemokine production. Additional clinical investigations on larger cohort groups and longer follow-up times are required to corroborate this possibility.

Before the COVID-19 pandemic belatacept was generally injected into patients at outpatient facilities. However, because there was a potential risk of these patients contracting nosocomial SARS-CoV-2 while attending the outpatient clinic during the epidemic, many centers implemented belatacept home infusion: a beneficial strategy for these patients [27].

Regarding the available data, we do not recommend postponing a belatacept infusion or switching to another immunosuppressive regimen. Instead, the organization of a dedicated infection-control protocol with stringent barrier precautions for patients that require regular outpatient infusions during the COVID-19 pandemic seems to be a good strategy.

## 3. Opportunistic Infections (OIs) and Overall Risk of Infection with Belatacept

### 3.1. Belatacept-Based Immunosuppressive Regimen Given to De Novo Transplant Recipients

The phase III BENEFIT (Belatacept Evaluation of Nephroprotection and Efficacy as First-line Immunosuppression Trial) study was a prospective multicentric randomized and controlled study. Within it, Vincenti et al. evaluated 354 adult KTx that received more intensive (MI) (173 patients) or less intensive (LI) (181 patients) regimens of belatacept versus CsA (173 patients). All recipients had received a kidney transplant from living or standard-criteria deceased donor. The initial follow-up was for 12 months.

The safety profile was excellent: indeed, although the number of OIs was not directly given, there was no statistically significant difference across the three groups in terms of cytomegalovirus (CMV) disease, BK virus (BKV) viremia, post-transplant lymphoproliferative disorder (PTLD), and tuberculosis [8]. The same cohort was followed for seven years. After seven years, the incidence of serious infections, of any grade of viral infection or any grade of fungal infection per 100 person-years (p-y) of treatment were the following: serious infections developed in 10.7 p-y for LI belatacept (*n* = 226) and 13.3 p-y for CsA (*n* = 221). [31].

Durbach et al. reported on the BENEFIT-EXT (BENEFIT—EXTended criteria donors) study. This 3-year, randomized, multicenter study included adults that had received a kidney transplant from an extended-criteria donor. A total of 578 patients were randomized: of the 543 that received a transplant, 184 received MI belatacept, 175 received LI belatacept. and 184 received CsA. After one year, the overall frequencies of infection and the incidences of bacterial, CMV, BK virus, and fungal infections were similar between the groups [9]. After seven years of follow-up, there were still 128 patients evaluable in the MI belatacept arm, 138 in the LI belatacept arm, and 108 in the CsA arm. Safety, in terms of infection, was quite good. Indeed, the incidence of serious infection per 100 p-y of drug exposure was similar across the treatment arms. The incidences of any grade of viral infection per 100 p-y of treatment were 20.98, 17.45, and 19.05 for MI belatacept, LI belatacept, and CsA, respectively. The corresponding values for the incidence of any grade of fungal infection per 100 p-y of treatment were 9.79, 6.93, and 11.00, respectively. There were no statistically significant differences between the groups [32].

In 2011, Ferguson et al. published a paper that reported on de novo KTx patients that received belatacept + mycophenolate mofetil (MMF) (27 patients), belatacept + sirolimus -SRL- (21 patients), or tacrolimus + MMF (28 patients). By month 12, there were no statistical differences between these groups in terms of OIs, viral or fungal infections [33].

### 3.2. Conversion from CNIs to Belatacept

In 2011, Rostaing et al. published the results from a phase II randomized prospective study in which they followed 173 KTx with stable graft function and that were receiving a CNI-based regimen. Between ≥6 and 36 months post-transplantation, they were randomized to be either switched to belatacept or continue with CNIs. All patients received background maintenance immunosuppression (MMF plus steroids). Eighty-four patients were switched to belatacept and 89 remained on a CNI-based regimen (30 received CsA and 59 received tacrolimus). At 12 months follow-up, there were no differences between the groups in terms of OIs [34].

Grinyó et al. published results from the same cohort after three years. They reported that exposure–adjusted rates for serious infections were similar between the belatacept group and the CNI group: i.e., 10.21 vs. 9.31 per 100 p-y, respectively [35].

In a retrospective multicenter European study published in 2018, 219 patients had been converted from CNIs to belatacept and were followed up for a median of 21 months. Belatacept was stopped for seven patients because of infections (three cases of cytomegalovirus [including two of CMV disease]; two had persistent BKV replication without BKV nephropathy; one had aspergillosis, and one patient had a bacterial infection). During the follow-up, there were 80 episodes of viral infection (36.5%) and 45 bacterial infections that required hospitalization (20.5%), of which most were pyelonephritis (8.5%). These results show a high rate of infection: 125 infections during 4803 months of exposure to belatacept, i.e., 31.2 events per 100 p-y. The rate of 34 OI events in 219 patients (10.5%), i.e., 8.5 OIs/100 p-y were mainly caused by CMV disease, varicella-zoster virus (VZV) infection, and *Pneumocystis jirovecii* [36].

Yazdi et al. reported on 78 KTx converted to belatacept. The median time until conversion was 242 days (~8 months) posttransplantation, with a median follow-up time of 433 days. Among the patients, 12 developed at least one infection after conversion (15%). The most notable infections were urinary-tract infection/pyelonephritis (*n* = 3), CMV viremia (*n* = 2), polyomavirus BK viremia (*n* = 2), osteomyelitis (*n* = 2), and cryptococcal pneumonia (*n* = 1), which all occurred by six months after conversion [37].

Gupta et al. (2020) reported on 53 adult patients with allograft dysfunction that underwent conversion to belatacept at a median of six months post-transplantation. At a median follow-up of 2.5 years, 64 adverse events had occurred during 88.5 p--y of belatacept exposure. Eight (15%) patients required hospitalization at post-conversion due to infection, with urinary-tract infections being the most common indication [38].

Brakemeier et al. (2016) looked at the use of belatacept as a rescue therapy. This retrospective study analyzed data from 79 adult KTx that were converted to belatacept. Patients had received a transplant on average 69.0 months prior to conversion and 44 had a GFR of <25 mL/min. Most grade ≥2 adverse events were for infection. The majority of reported viral (14/18, 77.8%) and bacterial infections (31/49, 63.3%) were grade 2. Two patients discontinued belatacept due to an infection: miliary tuberculosis, *n* = 1; CMV-related disease, *n* = 1. Of 19 reported infections, 12 were severe (urosepsis, *n* = 6; CMV, *n* = 2; *Pneumocystis jirovecii* pneumonia, *n* =1; miliary tuberculosis, *n* =1; phlegmon, *n* = 1; and *Candida* septicemia, *n* = 1) [39].

Two studies have looked specifically at the occurrence of OIs amongst patients that had received belatacept [11,40]. Bertrand et al. studied a French cohort of KTx converted from CNIs to belatacept at either early post-transplantation (<six months) or after six months. During the study period, 280 patients (mean ± SD age 56.6 ± 14.6 years) were switched from CNIs to belatacept at 10.4 months (median) after KTx: of these, 108 patients (38.6%) had early conversion and 172 patients (61.4%) had later conversion. Overall, 42 OIs occurred in 34 patients (12.1%), on average at 10.8 ± 11.3 months after the switch. After 5128 months of belatacept treatment, an incidence of 0.008 OI/month exposure and 9.8 OI/100 p-y was observed. The most common OI was CMV disease in 18/42 (42.9%) and *Pneumocystis jirovecii* pneumonia in 12/42 (28.6%). Two patients presented with progressive multifocal leucoencephalopathy, and two patients developed cerebral EBV-induced PTLDs. An OI led to death in 9/34 patients (26.5%) and graft failure in 4/34 patients (11.8%). After an OI, belatacept was stopped in 25/34 patients (73.5%). In multivariate analyses, an eGFR rate of <25 mL/min/1.73 m^2^ on the day of the switch and the use of immunosuppressive agents before transplantation were associated with the occurrence of an OI. The investigators also found a higher rate of infection-related hospitalization (24.1 vs. 12.3/100 p-y, *p* = 0.0007) and a higher rate of OIs (13.2 vs. 6.7/100 p-y, *p* = 0.005) in the early conversion group [11].

These results were assessed in a more recent study that merged the cohort of Bertrand et al. (*n* = 14 centers) with ours followed at Grenoble Hospital (conversion from CNI to belatacept cohort). In this study, Bertand et al. reported on a total of 453 KTx, across 15 transplantation centers, who were converted from CNI- to belatacept-based therapy. The median follow-up time between KTx and conversion to belatacept was 19 [0.13–431] months, and the mean time of follow-up after belatacept conversion was 20.1 ± 13 months. Most patients, i.e., 332 (79.3%), were converted to belatacept at >six months post-transplantation. As compared to the initial cohort, our monocentric cohort of 173 patients differed regarding the following criteria: patients were converted to belatacept later after post-KTx (median time: 57 months [0.5–43] vs. 10 [0.1–273], *p* < 0.001), i.e., 92.5% patients were converted after 6-months vs. 61.4% before, *p* < 0.001. Rate of lymphopenia at conversion was lower (46.2% vs. 77.1%, *p* < 0.001). Regarding the immunosuppressive regimen before conversion to belatacept: in our monocentric cohort, less patients received steroids (20.2% vs. 86%, *p* < 0.001) and CsA (5.2% vs. 24%, *p* < 0.001), and more patients received tacrolimus (93.6% vs. 71.8%, *p* < 0.001). Additionally, mean eGFR at conversion was higher (40.7 ± 9 vs. 26.6 ± 15, *p* < 0.001). OIs occurred in 42 (9.3%) patients after converting to belatacept at a mean time of 14 ± 12 months post-conversion. Amongst OI patients, eight (19%) presented with two OI episodes during the follow-up.

The authors assessed the incidence of all OIs at earlier and later conversion. The incidences of CMV DNAemia, and CMV disease were greater in patients converted earlier compared to those converted later (31.6% vs. 11.5%; *p* < 0.001; and 11.6% vs. 2.4%, *p* < 0.001, respectively). The cumulative incidence of OIs was 6.5 OIs/100 p–y. In multivariate analyses, only an eGFR of <25 mL/min/1.73 m^2^ at conversion was significantly and independently associated with the development of OIs (hazard ratio (HR) = 4.7 (2.2–10.3); *p* < 0.001). The HR of steroid use at conversion to predict OIs was 2.1 (0.8–5.2) but did not reach statistical significance (*p* = 0.121) [20]. A major limitation of these two studies was the absence of a control group receiving standard immunosuppressive therapy [11,40].

### 3.3. Expert Opinion

The cornerstone risk for an OI is the type of immunosuppressive regimen. Most studies on this subject have assessed the risks of infection based on a CNI-based regimen. The global risk related to tacrolimus being associated with severe infection is similar to that for CsA [22]. However, very few studies have focused on the risks associated with infection while on belatacept therapy. The rate of OIs after kidney transplantation varies in the literature from 10 to 31 infection events/100 p–y [9,11,41]. However, this rate should be interpreted with caution because it depends on the definitions given. Based on the data from early phase II and III studies, there is no evidence for an increased risk of OIs in patients on a belatacept-based immunosuppressive regimen. Thus, the rate of acute viral infections in the study by Rostaing et al. was higher than in the BENEFIT study (22.8% versus 13.3%), but the adverse event profile in this study may be a consequence of the older age of their cohort (53.8 vs. 45.3 years) and the longer time spent on immunosuppression (69.0 vs. 19.4 months). Darres et al. have confirmed these results [36].

Two recent studies have focused directly on OIs in a population of KTx that were converted from CNI to belatacept. Bertrand et al. reported an incidence of OIs of 9.8/100 p-y in those converted to belatacept, with a predominance of CMV and *Pneumocystis jirovecii* infections. Death occurred in 6.4% of patients and graft loss in 14.3% [11]. In the pooled cohort of belatacept-converted recipients, death occurred in 4.8% of patients and graft loss in 9.3% [40]. These data are much lower than the above data for death/graft survival and are very reassuring regarding conversion from CNI to belatacept-based therapy after KTx. By comparing the two pooled cohorts, the differences may be explained by the reduced risk of an OI within the whole cohort, i.e., patients from the second cohort (*n* = 173) were converted significantly later at post-KTx, had better eGFR at conversion, were less lymphopenic at conversion, received more frequent antithymocyte globulins (ATG) therapy at induction, had tacrolimus at conversion, and received less steroids and CsA at conversion [40]. The major risk factors for OI after belatacept conversion were a patient’s age, low graft function (<25 mL/min/1.73 m^2^), and the immunosuppressive therapy used before KTx (either for a failed previous transplant or for treating the pathology that led to end-stage kidney disease) [40]. The lower the eGFR, the higher the rate of OIs.

In all these studies, OIs were found to profoundly impact patients’ survival, although the data for non-death-censored graft survival were more heterogeneous. As for early conversion, even though graft function was improved, this strategy had greater risks regarding graft survival and the rate of OIs after an early switch compared to a later switch.

Nevertheless, clinicians need to be aware of the risks of such a strategy for patients with very low graft function. Monitoring and prophylaxis against OIs, mainly for CMV and Pneumocystis jirovecii, must be considered [11,40]. The role of belatacept is still unclear in this population of patients with multiple risk factors for infection and it is difficult to ascertain the direct role of belatacept in the occurrence of infections compared with the global immunosuppressed state of these patients.

In conclusion, as belatacept is today mainly used as a rescue strategy, there is a trend towards a higher occurrence of OIs, particularly after early conversion, in patients with very low graft function. In this context, clinicians need to be cautious if KTx present with fever and an unexplained inflammatory syndrome. In patients converted later to belatacept, the risk of an OI seems to be lower and the infection rate is then comparable to that reported in the literature. It has to be emphasized that the studies reporting a greater risk of OI in belatacept-treated patients have been retrospective non-controlled studies and where belatacept has been used as a rescue therapy. Case-control studies that analyze OIs in more homogenous cohorts of belatacept-treated patients, when used as a rescue therapy, could help clinicians draw firmer conclusions regarding the role of belatacept in the occurrence of severe infectious complications after conversion.

## 4. Belatacept and Viral Infections

### 4.1. Belatacept, CMV Infection, and Disease

#### 4.1.1. Belatacept and De Novo KTx

In the BENEFIT study, there were no differences between the groups in terms of CMV infection at 12 months: i.e., 13 (6%) with MI belatacept, 17 (8%) with LI belatacept, and 19 (9%) with CsA [8]. In addition, after seven years, there was no difference in terms of serious infectious viral disease. CMV infection developed in 1.4 p-y with MI belatacept (*n* = 219), 1.1 p-y for LI belatacept (*n* = 226), and 0.8 p-y for CsA [31]. Similarly, in the BENEFIT-EXT study, there was no difference between groups in terms of CMV infection at 12 months: 12 (7%) for MI belatacept, 14 (8%) for LI belatacept, and 12 (7%) for CsA [9]. At seven years, the cumulative incidence, adjusted per 100 p-y of treatment, was 2.2 in the MI belatacept group, 1.94 in the LI belatacept group, and 1.71 in the CsA group (not statistically significant) [32]. In the study by Fergusson et al., there was no statistically significant difference between the groups in terms of CMV infection (*n* = four total; *n* = 1 each for belatacept-MF and belatacept-SRL, and *n* = 2 for tacrolimus (TAC)-MMF) [33]. In all these studies, the rates for positive CMV viremia were not provided. In addition, it should be noted that the severity of these infections was not indicated [8,9,31,32,33].

In June 2014, Basil et al. reported on a single-center study that compared the incidence of viral infections, notably CMV, EBV, BKV, and JC virus (JCV), in de novo KTx that had received either belatacept-based or CsA-based immunosuppression. A total of 62 patients were included: 42 were randomized into the belatacept regimen (group 1: either more intensive or less intensive), and 20 into the CsA (group 2) regimen. The CMV donor (D) and recipient (R) status, notably D+/R+, D+/R−, D−/R+, and D−/R−, the number of patients on CMV prophylaxis, and the duration of prophylaxis were comparable between the groups. The incidence of CMV infection was 47% in group 1 and 45% in group 2 (*p* = NS). Primary infection rates in D+/R− CMV-status patients were 54% in group 1 and 45% in group 2 (*p* = NS). Seventy-six percent of CMV seropositive recipients in group 1 and 65% from group 2 had reactivation of CMV (*p* = NS) [42].

A more recent study addressed specifically the CMV risk in those patients treated with belatacept soon after transplantation. They focused on 168 de novo KTx with a high risk for CMV, i.e., donor CMV seropositive/recipient CMV seronegative, and looked at outcomes after KTx. Of these, 104 were treated with belatacept-based and 64 received tacrolimus-based therapy. They compared these patients to 140 de novo KTx at low risk for CMV infection (99 received belatacept-based and 41 received tacrolimus-based therapy). Overall, there were no significant differences in the patients’ characteristics according to CMV-risk. Among CMV high-risk patients, there was a non-statistically significant trend towards lower graft survival in patients treated with belatacept compared to those that received tacrolimus, with 1-, 3-, and 5-year graft survival rates of 95.0 [90.8; 99.4]%, 89.7 [83.9; 96.0]%, and 85.7 [78.8; 93.3]% among patients treated with a belatacept-based regimen compared to 100.0%, 98.0 [94.1; 100]%, and 91.7 [83.0; 100]% among patients treated with a tacrolimus-based regimen. After adjustment for the patients’ characteristics and donor type, the hazard ratio for a poor outcome in CMV high-risk recipients treated with a belatacept-based regimen was 1.63 [0.78; 3.4] compared to patients treated with a tacrolimus-based regimen, which was mostly driven by a higher risk of graft loss. The risk of a poor outcome did not differ based on immunosuppression regimen in CMV low-risk patients (hazard ratio [HR] belatacept: 0.97 [0.31; 3.00]). Similarly, there were no significant differences in patients’ survival between belatacept-based and tacrolimus-based regimens in both CMV high- and low-risk patients. CMV high-risk patients treated with a belatacept-based regimen were more likely to develop CMV viremia during the first two years posttransplant with a 2-year cumulative incidence of CMV viremia of 50.0 [49.5–50.5]% among the belatacept-based regimen compared to 34.4 [33.7–35.1]% among the tacrolimus-based regimen (*p* = 0.47).

Among patients with at least one episode of viremia, maximum viral load did not significantly differ between belatacept-treated patients (median [interquartile range] viral load = 136,000 [37,400; 508,000] copies/mL) and tacrolimus-treated patients (median [interquartile range] viral load = 44,950 [22,850; 338,250] copies/mL), *p* = 0.11. However, it took longer for belatacept-treated patients to achieve virus clearance, as demonstrated by a higher number of positive viral loads and a higher area under the curve. Furthermore, the authors found a higher rate of CMV resistance to ganciclovir among belatacept-treated patients (22.1%) compared to patients in the tacrolimus group (1.6%) (*p* = 0.001). This resulted in a greater use of second-line antiviral drugs in those receiving a belatacept-based regimen (20% of viremic patients) compared to those on a tacrolimus-based regimen (5%) (*p* < 0.001) [43].

#### 4.1.2. Belatacept in the Setting of Conversion, i.e., after CNI-Based Treatment

In a phase II study by Rostaing et al., at 1-year post-randomization, CMV infection developed in two patients from the belatacept and CNI groups. In addition, asymptomatic CMV viremia occurred in one patient from the belatacept group and none from the CNI group [34]. After three years of follow-up, the cumulative incidence of selected adverse events in terms of CMV infection were, respectively, 1.27 and 0.00 per 100 p-y of treatment exposure in the belatacept and CNI groups, respectively, thus showing a trend towards more CMV infection in the belatacept groups, although this was not statistically significant [35]. The severity of the infection was not reported.

In a retrospective study by Darres et al., among 219 KTx converted from CNI-based to belatacept-based therapy, the authors found that 23 (10.5%) had CMV replication with six primary CMV infections and 17 CMV reactivations. In addition, seven patients suffered from CMV disease (2.7%), and three patients had to stop belatacept treatment because of CMV infection, which included two CMV diseases. However, the severity of the CMV disease was not reported in the study [36]. In a review by Yazdi et al., only two of 78 KTx converted from CNI-based to belatacept-based therapy had positive CMV viremia, without signs of infection [37]. In a study by Gupta et al., six of 53 (11.3%) converted KTx developed CMV-associated disease: of these, three were at high risk for CMV, i.e., the donor was CMV seropositive, the recipient was CMV seronegative, thus putting them at risk for primary CMV infection [38].

In the study by Bertrand et al., patients were converted from CNI-based to belatacept-based immunosuppression at either early or late after kidney transplantation: CMV disease was more frequent after early conversion to belatacept (13/18 patients, 72.2%), and occurred at 10.8 ± 10.1 months (range 0.4–39.7) after the switch. In high-risk D+/R− patients, CMV disease occurred as a primo-infection in 9/18 cases (50%). In intermediate-risk patients (D+/R+ and D−/R+), CMV disease occurred in 7/18 cases (38.9%). CMV disease occurred in two (11.1%) low-risk patients (D−/R−). In high-risk CMV patients, CMV disease occurred mainly after early conversion (8/9, 88.9%) compared with intermediate-risk patients (3/7, 42.9%). Patients presenting with CMV disease did not receive anti-CMV prophylaxis. In eight cases, CMV disease presented with multi-organ involvement and, importantly, as this was an exceptional finding in KTx, two cases of CMV chorioretinitis were diagnosed. Four patients with CMV disease after conversion died (4/18, 22.2%), but in 3/4 cases CMV disease was associated with another OI (two of *Pneumocystis jirovecii* pneumonia and one of cerebral toxoplasmosis) [11].

These concerning results were considered in a second study by Bertrand et al., in which they merged their data with ours: i.e., 173 patients from a monocentric cohort in Grenoble Hospital [40]. After six months of belatacept treatment the incidence of CMV disease was lower, i.e., 2.8/100 p-y versus 2.11/100 p-y, as reported by Bertrand et al. [11]. In the pooled data from the two cohorts, the numbers of patients with positive CMV viremia were 74 (16.9%), i.e., 37 (31.6%) in the early conversion group and 37 (11.5%) in the late conversion group (*p* < 0.01). CMV disease occurred in 22 patients (4.8%): 14 in the early conversion group (8.6%) and eight (2.4%) cases in the late-conversion group (*p* < 0.01) [40].

In the study by Karadkhele et al., patients at high-risk for CMV and treated with a belatacept-based regimen experienced a higher incidence of CMV viremia, with half of the patients developing viremia of >1000 copies/mL within two years after transplantation and a longer time needed to clear the virus. Moreover, they found a trend towards a higher risk of graft loss in these high-risk patients although this did not reach statistical significance [43].

Chavarot et al. analyzed the characteristics of CMV disease after conversion to belatacept [44]. Propensity-score matching was used to compare the incidence of CMV disease in belatacept- and CNI-treated KTx. The authors focused on patients that received belatacept for more than six months to ensure significant exposure to the drug. A total of 223 KTx (aged 59.2 [range: 45.4–68.5] years), converted to belatacept and exposed to belatacept for more than six months, were included. CMV disease occurred in 40/223 patients (17.9%) after a median of nine months (0.23–54.8) from conversion and at 29.5 months (4.5–104.1) after transplantation. The cumulative incidence of CMV disease was 6.6 per 100 p-y. A total of 181 patients (81.2%) treated with belatacept were matched with 181 patients treated with CNI-based immunosuppression. After conversion/matching, 32/181 belatacept-treated patients (17.7%) experienced CMV disease, whereas 5/181 patients (2.8%) experienced CMV disease in the control group. Overall, the cumulative incidence of CMV disease over time was 6.33 per 100 p-y (95% CI [4.474–8.946]) in the belatacept group and 0.91 per 100 p-y (95% CI [0.377–2.175]) in the control group, corresponding to a sevenfold higher risk of CMV disease with belatacept treatment (*p* < 0.0001). Multivariate analysis identified D+/R− CMV serostatus (HR: 7.703, 95% CI [1.693–35.047], *p* = 0.0220), age at conversion (HR per year: 1.032, 95% CI [1.006–1.058], *p* = 0.0164), and eGFR at conversion (HR: 0.973, 95% CI [0.948–0.998], *p* = 0.0355) as independent risk factors for CMV disease in belatacept-treated patients [44].

Importantly, as in the first study by Bertrand et al. [11], the vast majority of cases with initial CMV disease and receiving belatacept had tissue-invasive disease. The most common presentation of CMV disease was gastrointestinal in 32/40 patients (80%) and included severe life-threatening hemorrhagic colitis in 4/40 patients (10%) and upper gastrointestinal-tract involvement (esophagitis or duodenitis) in 5/40 patients (12.5%). Other clinical presentations were hepatitis in 3/40 patients (7.5%) and bilateral retinitis in 2/40 patients (5%). Twenty-one recurrent episodes of CMV disease occurred in 13/40 patients (32.5%), at a median time of 3.3 months (2.2–5.6) after the first episode. Five of 13 of these patients (38.5%) experienced multiple clinical recurrences (two recurrences in five patients, three recurrences in two patients, and four recurrences in one patient). Again, the majority of recurrent CMV diseases was tissue-invasive diseases with gastrointestinal involvement and developed in more unusual locations. Notably, resistance to valganciclovir was documented in three patients [44].

#### 4.1.3. Expert Opinion

Cytomegalovirus (CMV) replication and/or infection is a common complication following KTx. Late CMV infection remains a significant problem [45]. In a recent study, Leeaphorn et al. (in the US), demonstrated that CMV infection still affects both patient- and graft-survival rates despite the use of prophylactic treatment [46]. However, the results from the BENEFIT and BENEFIT-EXT studies are reassuring regarding the risk of CMV infection [8,9]. It has to be noted that, in those studies, CMV viremia was not systematically followed and only data from complicated CMV infections were collected (see Table 1).

Recent results suggest concern for patients that receive belatacept as an early conversion therapy. Bertrand et al. and Chavarot et al. [11,44] report on how CMV disease was particularly aggressive in a multisystemic way and/or had atypical localizations in KTx. CMV disease developed mainly in patients at high- and/or intermediate-risk for CMV, but also in low-risk patients. CMV serostatus, age at conversion, and eGFR at conversion were independent risk factors for CMV disease in belatacept-treated patients [11,44]. In these two cohorts, the proportion of patients treated with steroids (mostly 10 mg per day) was high (respectively, 100% and 86% in the Chavarot et al. and Bertrand et al. cohorts). This could explain why the rate of CMV infection was lower in the second pooled cohort (presented in the second paper by Bertrand et al.) [40]: i.e., 20.2% of patients were weaned off steroids.

Due to the relatively high rate of CMV infection observed in recent studies, monitoring for CMV replication under belatacept-based therapy seems to be required. Administration of CMV prophylaxis for a few months at the time of conversion, especially for high-risk patients (D+/R−), is recommended.

We do not recommend the avoidance of belatacept in CMV high-risk recipients, as ~50% of these patients did not develop CMV infection. However, it seems reasonable to extend the duration of CMV prophylaxis, e.g., to 12 months, for patients at high risk for CMV infection even though evidence is currently lacking to support the benefit of such an approach. We suggest to treat asymptomatic CMV viremia and, if it still persists after three weeks on appropriate therapy, to look for CMV resistance and to withhold belatacept therapy. In the case of CMV disease belatacept should be stopped. Finally, we recommend not converting a patient from CNI-based to belatacept-based therapy if he/she has presented recently with CMV disease without CMV seroconversion [43].

The underlying mechanisms that lead to increased rates and a higher burden of CMV viremia with belatacept remain to be investigated. Belatacept interrupts the CD28-B7 costimulatory pathway, which plays a critical role in the development of primary T-cell responses but is less important for recall immune responses [47]. Indeed, belatacept was less effective than tacrolimus in reducing CMV-specific cytokine release from peripheral blood mononuclear cells in CMV-positive patients [48].

### 4.2. Belatacept and BKV Viruria, Viremia, and Nephropathy

#### 4.2.1. Belatacept and De Novo KTx

In the BENEFIT and BENEFIT-EXT studies there are few data concerning BKV infection. In the BENEFIT study, at 12 months, there was no statistically significant difference between the groups in terms of BKV infection: 10 patients (5%) in the MI belatacept group, three patients (1%) in the LI belatacept group, and nine (4%) in the CsA group [8]. In the BENEFIT-EXT study, at 12 months, the authors report that there was no statically significant difference between the groups in term of BKV infection, but the data were not shown [9]. In the study by Fergusson et al., BK virus infection occurred in two patients: one that received belatacept-MMF and the other TAC-MMF [33].

Bassil et al. reported that 4.7% of belatacept-treated patients developed BKV nephropathy compared to 5% that were treated with CsA (*p* = NS). Median time from transplantation until detection of the first positive BKV DNAemia was eight months in the belatacept group compared to four months in the CsA group (*p* = NS). The number of positive BKV viremias over the three years was similar across the groups [42].

#### 4.2.2. Belatacept in the Setting of Conversion, i.e., after CNI-Based Treatment

In a phase II study reported by Rostaing et al. BK-virus infection occurred in three patients in the belatacept group (4%), with one case of polyomavirus-associated nephropathy (1%) at 12 months. None of the patients treated with CsA had a BKV infection during follow-up [34]. At three years, the cumulative incidence rates of BKV infection were, respectively, 0.85 (belatacept) and 0.00 (CNIs) per 100 p-y of treatment (*p* = NS) [35]. In the study by Darres et al., two patients receiving belatacept developed BKV nephropathy (0.9%) and 19 patients developed BKV viremia (8.7%) [36].

Gupta et al. noted BKV viremia in one patient (1.9%) [38]. Bertrand et al. focused on OIs in their study and considered polyomavirus-associated nephropathy as an OI, but not BKV viremia. However, there was no information on BKV viremia in their study, and no patients suffered from polyomavirus-associated nephropathy [11]. In the second study by Bertrand et al., only six patients developed BKV infection (2%) and none developed polyomavirus-associated nephropathy [40]. It should be noted that, in all these studies, there was no information on BKV viruria.

#### 4.2.3. Expert Opinion

In terms of BK viremia and polyomavirus-associated nephropathy, the safety profile for belatacept is relatively good. There are no data to suggest an increased risk of BKV infection compared to a CNI-based immunosuppressive regimen.

The prevalence of BKV viruria and BKV viremia in renal-transplant recipients is ~40% and 12%, respectively [49]. BK virus nephropathy, which affects about 8% of recipients, manifests as an asymptomatic gradual rise in serum creatinine levels and results in allograft loss or permanent dysfunction in 40–60% of cases [50]. Since no effective antiviral exists, the only established therapy remains pre-emptive reduction of immunosuppression, but this entails increased risks of alloimmunity [51,52,53,54].

We recommend monitoring for BKV DNAemia and DNAuria every six months while on belatacept-based therapy. There is no consensus on an adequate strategy for cases of BKV infection or nephropathy in a patient treated by belatacept. We do not suggest stopping belatacept in cases of BKV infection or nephropathy, but to reduce or stop immunosuppressive co-medication, i.e., which in most cases is mycophenolic acid. We also suggest conversion from MMF/MPA to the mammalian target of rapamycin inhibitor (mTORi). Indeed, mTOR inhibitors suppress BKV replication in vitro, whereas tacrolimus activates virus production [55]. Finally, as it has been shown that intravenous immunoglobulins (IVIGs) contain high amounts of neutralizing BKV-specific antibodies in vitro [56], we suggest adding IVIGs in the event a belatacept-treated patient presenting with BKV infection. However, IVIGs should not be given at the same time as belatacept infusion, i.e., IVIGs may inhibit Belatacept by complexing it.

### 4.3. Belatacept, JCV Viremia, and Progressive Multifocal Leukoencephalopathy (PML)

#### 4.3.1. Belatacept and De Novo KTx

There were no cases of PML in the BENEFIT study or in Ferguson et al.’s study [8,33]. In the BENEFIT-EXT study, a single case of PML was reported after month 12 in a patient that received belatacept (MI), basiliximab, mycophenolate mofetil, and corticosteroids [9]. Unfortunately, there was no information about JCV viremia in these studies.

In a study by Bassil et al., over a 3-year period, five patients developed JCV viremia: all were within the belatacept group, although there was no statistical difference between the groups regarding the number of positive JCV viremias compared to the number of JCV PCRs performed in the first, second, and third years. None of the patients with JCV DNAemia developed PML [42].

#### 4.3.2. Belatacept in the Setting of Conversion, i.e., after CNI-Based Treatment

A phase II study (Rostaing et al.) showed no cases of PML at 12 months or at three years [34,35]. In a European retrospective study, Darres et al. reported one episode of JCV replication (0.5%) without PML [36]. Bertrand et al. reported that two patients presented with PML (assessed by MRI) and positivity for the JC virus in cerebrospinal fluid. These two PML cases occurred in patients that had an early conversion to belatacept (switch 2.7 and 2.1 months post-KTx) with PML occurring at 56.2 and 28.8 months post-conversion. One of the two patients died [11].

#### 4.3.3. Expert Opinion

There are few data concerning JCV and belatacept treatment: however, the data reported are reassuring. JCV viremia seems to be a reflection of over immunosuppression. Indeed, Bassil et al. report that all patients with JCV viremia had other viral infections: four cases of CMV with CMV viremia, four cases with EBV viremia, and one case with mixed EBV, CMV, and BKV viremias [42]. To our knowledge only three cases of JCV replication have been reported so far; two of these presented without PML [11]. The exact role of belatacept in this disease is still unknown, as are the risk factors to KTx [57]. Discontinuation of belatacept in the context of PML is highly recommended. We do not recommend monitoring for JCV DNAemia routinely in belatacept-treated patients.

### 4.4. Belatacept, EBV Viremia, and Posttransplant Lymphoproliferative Disorder (PTLD)

#### 4.4.1. Belatacept and De Novo KTx

In the BENEFIT study, one patient in the MI belatacept group, two in the LI group, and one in the CsA group had developed PTLD by month 12 [8]. At seven years, only one other case of PTLD, occurring between 12 and 24 months after transplantation, was reported in the group assigned to the more-intensive belatacept regimen (incidence rate, 0.1 cases per 100 p-y) [31].

In the BENEFIT-EXT study, one patient (0.5%) in the MI belatacept group and two patients (1%) in the LI group developed PTLD during the 12-month period, and one additional patient in each belatacept group developed PTLD after month 12. Four of the five cases involved the central nervous system, and two of five (both the post month-12 cases) had concomitant CMV disease. No patients receiving CsA developed PTLD. Three of the five PTLD patients had negative EBV serology at pretransplant. No patients that developed PTLD in this study had taken lymphocyte-depleting therapy. As of March 2009, three of the five PTLD patients had died (*n* = 2 MI; *n* = 1 LI) and one LI belatacept patient was alive but had lost the allograft after a nephrectomy [9]. At seven years, nine patients experienced PTLDs: of these, four were EBV positive. Incidence rates per 100 p-y of exposure in EBV-seropositive patients treated with MI belatacept, LI belatacept, and CsA were 0.12, 0.25, and 0.14, respectively. The corresponding values in EBV-seronegative patients were 1.71, 5.19, and 0.00, respectively. Of those patients that had PTLDs, five had primary central nervous system PTLDs (two cases of MI belatacept; three of LI belatacept) and seven had died (three cases of MI belatacept; four of LI belatacept) [32]. In the study by Ferguson et al. there were no cases of PTLD: no information was provided on EBV viremia [33].

In a study by Bassil et al., EBV donor and recipient serostatus (i.e., D+/R+, D+/R−, D−/R+, and D−/R−) were similar in both groups. Primary infection occurred in two of the three EBV D+/R− belatacept-treated patients and two of the four EBV D+/R− CsA-treated patients (*p* = NS). Rates of EBV reactivation numerically differed between both groups: 78% in belatacept-treated vs. 50% in CNI-treated patients (*p* = 0.056). Rate of EBV infection (primary infection or reactivation) was statistically higher in the belatacept group: i.e., 76% vs. 50% (*p* = 0.047). Over the 3-year period, there were two cases of PTLD in belatacept patients (EBV-seropositive patients) and none in the CNI group. Both cases of PTLD were located within the central nervous system; both were EBV-related [42].

#### 4.4.2. Belatacept in the Setting of Conversion, i.e., after CNI-Based Treatment

In a phase II study by Rostaing et al. (follow-up by Grinyó et al.,) there were no cases of PTLD (21); there was no information on EBV viremia [34,35]. In the study by Darres et al., two patients developed PTLDs in the central nervous system: both cases were EBV seropositive and there was detectable EBV replication in the blood [36].

In a study by Yazdi et al., one patient developed EBV-associated PTLD at 13 months after conversion. This patient had been converted at less than 1-year posttransplantation and had not experienced rejection at pre- or post-conversion. Belatacept was discontinued at the time of diagnosis and the patient was alive with a functioning allograft at the last follow-up [37].

In the first study by Bertrand et al., two EBV seropositive patients developed cerebral EBV-induced post-transplant PTLD: this occurred in both cases after late conversion to belatacept (149.7 and 124.2 months post-KT) at 27.6 and 4.1 months after conversion [11]. In the second study by Bertrand et al., there were no additional cases of PTLD. EBV DNAemia rates were higher in patients converted later compared to those converted earlier (42.7% vs. 14.8%, *p* < 0.001). Sixteen patients with EBV DNAemia developed an OI (12%), whereas 117 did not (88%). There was no statistical association between OIs and positive-EBV DNAemia (*p* = 0.62). EBV DNAemia had an incidence of 17.3 events/100 p-y [40].

#### 4.4.3. Expert Opinion

In adult solid-organ recipients (SOTs), PTLD has been reported in up to 2.3% of kidney transplants [58]. Over a 10-year period, the risk of PTLD in KTx was 12-fold higher than in the general population [59]. It is associated with a reported mortality of 40–60% [60].

After the results from the BENEFIT and BENEFIT-EXT studies and the concerns regarding PTLD, belatacept-based therapy is implemented only in EBV seropositive KTx [61]. The significance of EBV DNAemia in EBV-seropositive KTx is still a matter of debate [41,62]. Martin et al., in a prospective cohort of 383 consecutive de novo KTx, found that EBV reactivation during the first-year post-transplantation was frequent and reflected over immunosuppression [62]. There are no data that show a clear association between OIs and positive EBV DNAemia [40]. We recommend monitoring for EBV DNAemia at least once a year in belatacept-treated patients.

Overall, the incidence of PTLD in belatacept-treated patients is 0–4%, which is comparable to that found in patients receiving a CNI immunosuppressive regimen [62]. If PTLD is diagnosed, we recommend stopping belatacept treatment and reducing the immunosuppressive burden.

### 4.5. Belatacept and HIV, HBV, and HCV Infections

#### 4.5.1. Belatacept and HIV Infection

##### Belatacept in De Novo KTx

To date there are no data on belatacept use in de novo HIV-positive KTx.

##### Belatacept in the Setting of Conversion, i.e., after CNI-Based Treatment

Despite improvements in HIV+ treatments, KTx outcome rates from infectious complications and acute rejections remain higher than for those in non-HIV KTx. Santeusanio et al. reported on a retrospective study that described short-term outcomes following conversion from CNI to belatacept in a cohort of 10 HIV+ patients. With a median follow-up of 3.3 years, eight patients remained on belatacept therapy, and all patients were alive with functioning allografts. All patients maintained undetectable HIV-1 viral loads at last follow-up and no patient required modifications to antiretroviral therapy (ART) to maintain viral suppression. One patient developed *Pneumocystis* pneumonia and Kaposi sarcoma following conversion but was responsive to standard medical therapy. No other major side effect was noted [63].

El Sakhawi et al. performed a controlled study on 12 HIV-positive KTx that were converted from CNI to belatacept (belatacept group), of which three had early switches. The control group comprised HIV+ KTx receiving CNI-based therapy. At three and 12 months after kidney transplantation, in belatacept-treated patients, allograft survival was 92%. After 12 months of belatacept treatment, the CD4^+^ T-cell count was 318 (150–608)/mm^3^ and HIV viral load rebound was identified in two (17%) patients that also admitted non-adherence to ART for a couple of weeks. However, their viral load decreased as soon as ART was reinitiated. No significant difference in the patient (*p* = 0.38) and allograft survival (*p* = 0.36) was detected between the belatacept group and the control group. Three belatacept-treated patients (25%) developed opportunistic infections within 12 months post-conversion: one had disseminated tuberculosis with macrophage-activation syndrome that incurred belatacept disruption at three months after conversion and at 12 months after transplantation, one had CMV colitis that developed 57 months after conversion and 60 months after transplantation, and the third had chronic norovirus-related diarrhea but with a good outcome after reducing doses of immunosuppressants. Another two patients (17%) presented with CMV viremia without developing CMV disease at one and 27 months after the switch. Two patients (17%) presented with BK viremia at one and 25 months after the switch. In the control group, the incidences of OI [*n* = 1 (5%); *p* = 0.28], CMV viremia [*n* = 4 (33%); *p* = 1.00] and BK viremia [*n* = 2 (10%); *p* = 0.62] did not significantly differ from their counterparts [64].

##### Expert Opinion

There are few studies regarding belatacept use in HIV+ KTx. In the two published studies, allograft survival and eGFR were good. Incidence of acute rejection and opportunistic infection was acceptable, HIV disease remained under control after belatacept treatment, and CD4+ and CD8+ T-cell counts remained stable [63,64]. We recommend performing conversion from CNI to belatacept at least six months after transplantation in HIV+ KTx. Screening for infections should be more frequent after conversion, especially in patients that have had a pre-transplantation or pre-conversion opportunistic infection.

#### 4.5.2. Belatacept: HBV and HCV Infection

To our knowledge, only one case of hepatitis B reactivation has been described in association with the use of belatacept after kidney transplantation. The patient had been treated by belatacept in a de novo setting. Two years after transplantation, severe acute hepatitis occurred with a tenfold increase in AST and ALT levels and a twofold increase in bilirubin, but no signs of hepatic failure. Treatment with MMF was interrupted, and treatment with entecavir was started. Belatacept was not discontinued. The outcome was favorable after two months of antiviral treatment. HBV-DNA became undetectable after nine weeks of treatment, and liver tests completely normalized [65].

##### Expert Opinion

Chronic HBV infection is not a major concern regarding belatacept use in kidney transplantation providing that the patient is HBV DNA negative on antiviral therapy. HCV seropositive KTx should be HCV RNA(-). We recommend avoiding belatacept as a de novo immunosuppressive treatment or in an early conversion setting for recipients whose donor is HCV positive. In HCV(+)/RNA(+) KTx we recommend curing an HCV infection before conversion to belatacept. For HBV(+) KTx receiving antiviral drugs, we recommend monitoring HBV viremia before and then every six months after conversion to belatacept.

## 5. Belatacept and Fungal Infections

### 5.1. Belatacept and Overall Risk of Fungal Infection

#### 5.1.1. Belatacept and De Novo KTx

In the BENEFIT-EXT study, at 12 months, six serious fungal infections were reported: one case each of *Pneumocystis jiroveci* pneumonia, esophageal candidiasis, and fungal respiratory-tract infection in the MI belatacept group, one case of *Pneumocystis jiroveci* pneumonia in the LI belatacept group, and one case each of fungal infection and fungal pneumonia in the CsA group [9]. After seven years, the corresponding values for the incidence rates of any grade of fungal infection per 100 p-y of treatment exposure were 9.79, 6.93, and 11.00 [32]. In the study by Ferguson et al., fungal infections occurred more often in the belatacept--MMF group (*n* = 5) but were generally mild skin infections that responded to treatment, with the exception of one serious event of cryptococcal meningitis, but that resolved with treatment and did not lead to treatment discontinuation [33].

#### 5.1.2. Belatacept in the Setting of Conversion, i.e., after CNI-Based Treatment

In a phase II study by Rostaing et al., at 12 months, fungal infections were more frequent with belatacept (11 patients [13%]) than with CNI therapy (three patients [3%]). Infections mostly consisted of mild or moderate skin or oral infections. None were classified as serious or resulted in discontinuation of the study drug [34]. At three years, there were more any-grade fungal infections in the belatacept group (9.73 per 100 p-y) than in the CNI group (2.58 per 100 p-y); the most common types of fungal infection were onychomycosis and tinea versicolor [35]. In the study by Darres et al., among 219 patients, eight developed a fungal infection (3.7%): three cases of Pneumocystis jirovecii (1.4%), two of aspergilloses (0.9%), two Candida infections (0.9%), and one Cryptococcus infection (0.5%) [36].

Gupta et al. reported on 53 patients of which three (5.7%) developed Cryptococcus [38]. In the study by Brakemeier et al., among 69 patients, one developed Pneumocystis jirovecii pneumonia and one developed Candida septicemia [39].

The two studies by Bertrand et al. focused on OIs. Therefore, there is no information regarding mild to moderate fungal infections. In the first study, nine patients developed Pneumocystis pneumonia and one patient developed Aspergillus fumigatus colitis [11]. In the second study, Pneumocystis pneumonia was 1.6/100 p-y and Aspergillosis pneumonia was 0.2/100 p-y [40].

#### 5.1.3. Expert Opinion

There is a trend towards more fungal infections in belatacept-treated patients in a conversion setting, but these infections are mostly of mild to moderate severity and have only very rarely necessitated treatment to be stopped. Severe fungal infections are rare, apart from Pneumocystis pneumonia, which we discuss below. To our knowledge, no study has specifically focused on fungal infections associated with belatacept. Published studies have not described the characteristics of the patients that have developed fungal infections (excepted for Pneumocystis).

Overall, fungal infections are not a major issue for patients on a belatacept immunosuppressive regimen, excepted for Pneumocystis pneumonia.

### 5.2. Belatacept and Pneumocystis Jiroveci Infection

Belatacept and Pneumocystis Jiroveci Infection could be seen in Table 2.

#### 5.2.1. Belatacept Given to De Novo KTx

In the BENEFIT study, at 12 months, and in Ferguson et al.’s study, none of the patients developed *Pneumocystis* pneumonia [8]. In the BENEFIT-EXT study, at 12 months, one patient from each group developed *Pneumocystis* pneumonia (MI belatacept, LI belatacept, and CsA) [9].

#### 5.2.2. Belatacept in the Setting of Conversion, i.e., after CNI-Based Treatment

In a phase II study (Rostaing et al.), no patient developed *Pneumocystis* pneumonia at 12 months or at three years [34,35]. In the study by Darres et al., only three cases of *Pneumocystosis* (1.4%) were reported, but the conditions of specific prophylaxis after conversion were not specified [36].

In the first study by Bertrand et al., nine patients developed *Pneumocystis* pneumonia: it occurred after early conversion to belatacept in 6/12 cases (50%), at 13.9 ± 11 months (range 0.5–39.7) after the switch. Patients with *Pneumocystis* pneumonia were not receiving prophylaxis. Patients with *Pneumocystis* pneumonia had another OI (7/12 patients), mainly CMV disease (6/12). Mortality was higher in patients diagnosed with *Pneumocystis* pneumonia (4/12, 33.3%) [11]. In the second study by Bertrand et al., 13 patients developed *Pneumocystis* pneumonia (2.8%), of which six (4.9%) were in the early-switch group and seven (2.1%) in the later switch group (*p* = 0.057) [40].

#### 5.2.3. Expert Opinion

For KTx, several risk factors for *Pneumocystis* pneumonia have been described, such as escalation of immunosuppression, cytomegalovirus (CMV) infection, low eGFR, advanced age, and prolonged lymphopenia [66,67,68,69,70]. Among immunosuppressive regimens, rituximab and antithymocyte globulin (ATG) seem to especially increase the risk for *Pneumocystis* pneumonia [71].

Recently, Brakemeier et al. [72] tried to determine the risk factors for *Pneumocystis* infection after kidney transplantation in the modern era of immunosuppressive regimens. Patients after ABO-incompatible transplantation and those treated with belatacept had an increased risk for Pneumocystis jirovecii pneumonia (7.3% and 4.3%, respectively). However, patients treated with belatacept had other risk factors, such as older age, low eGFR, and lymphopenia, which may have contributed to this increased risk. In the two studies by Bertrand et al., early conversion and low eGFR were risk factors for developing *Pneumocystis* pneumonia. Several patients had concomitant OIs, mostly CMV disease [11,21].

The high occurrence of *Pneumocystis* pneumonia developing soon after KTx and after conversion to belatacept could be an argument for a direct role of belatacept in the development of this disease [11,21]. In this context, prophylaxis against *Pneumocystis* is recommended after an early switch from CNI to belatacept in KTx with very low graft function. The major problem remains the duration of prophylaxis.

## 6. Belatacept and Bacterial Infection

### 6.1. Belatacept and Overall Risk of Bacterial Infection

Belatacept and Overall Risk of Bacterial Infection could be seen in Table 3.

#### 6.1.1. Belatacept Given to De Novo KTx

In the BENEFIT study, at 12 months, the only serious adverse event (that occurred in >5% of patients) was urinary-tract infection, with no differences in prevalence between the groups: 54 (25%) in the MI belatacept group, 63 (28%) in the LI belatacept group, and 50 (23%) in the CsA group [8]. In the BENEFIT-EXT study, at 12 months, there was no difference between the groups in terms of urinary-tract infection (55 [30%] in the MI belatacept group, 57 [33%] in the LI belatacept group, and 62 [34%] in the CsA group) and bacterial pneumonia (respectively, eight cases [4%], seven [4%], and five [3%]) [9]. At seven years, the cumulative incidence rates for selected bacterial events, adjusted per 100 p-y of treatment, were similar between groups: urinary-tract infection, rates were 3.02, 3.62, and 3.54, respectively; pyelonephritis rates were 1.44, 0.69, and 1.83, respectively; pneumonia rates were 1.76, 1.50, and 1.41, respectively; bacteremia rates were 0.34, 0.11, and 0.63, respectively; the septic shock was 0.23, 0.22, and 0.75, respectively, in the MI belatacept, LI belatacept, and CsA groups, respectively [32].

In 2010, Grinyo et al. pooled the results from the BENEFIT, BENEFIT-EXT studies and the study of Ferguson et al. They found that the most common bacterial infection within each group was urinary-tract infection (MI: 34%; LI: 36%; CsA: 34%). The most common serious infections occurred with similar frequency between the groups and included urinary-tract infection (MI: 8%; LI: 8%; CsA: 10%), pneumonia (MI: 4%; LI: 3%; CsA: 4%), and pyelonephritis (MI: 4%; LI: 2%; CsA: 3%) [73].

#### 6.1.2. Belatacept in the Setting of Conversion, i.e., after CNI-Based Treatment

In a phase II study by Rostaing et al., at 12 months, few serious adverse events were reported. Serious bacterial events that occurred included pyelonephritis (two with belatacept, one with CNI), pyrexia (three with belatacept), and urinary-tract infection (two with belatacept) [34]. In the study by Darres et al., 45 patients (20.5%) suffered from a bacterial infection that required hospitalization, including 18 (8.2%) cases of pyelonephritis and 12 (5.5%) of pneumonia [36]. Gupta et al., among 53 patients, reported that four (7%) developed a surgical wound infection, 13 (24.5%) a urinary-tract infection, three (5.7%) bacteremia, and one (1.9%) developed *Clostridium difficile* colitis [38].

#### 6.1.3. Expert Opinion

Overall, the rate of bacterial infection was reasonable and comparable with the rates associated with a CNI-based immunosuppressive regimen. In general, bacterial infections were not a major issue for patients on a belatacept immunosuppressive regimen.

### 6.2. Belatacept and Risk of Tuberculosis (TB)

Tuberculosis infection is not a major issue for patients on a belatacept-based immunosuppressive regimen, excepted in endemic areas.

#### 6.2.1. Belatacept and De Novo KTx

In the BENEFIT study at 12 months and seven years, and in the study by Ferguson et al., no case of tuberculosis has been described [8,31,32,33]. In the BENEFIT-EXT study, tuberculosis was reported in four belatacept-treated patients; of which three cases occurred within an endemic area, of which two were from a single site. No patients in the cyclosporine group developed tuberculosis [9].

#### 6.2.2. Belatacept in the Setting of Conversion, i.e., after CNI-Based Treatment

In the phase II study by Rostaing et al. there was a single case of tuberculosis in the belatacept group. The patient, living in Mexico, had no prior history of tuberculosis. After successful treatment, the patient remained in the study and continued receiving belatacept [34]. In the studies by Darres et al., Yazdi et al., and Gupta et al. there was no case of tuberculosis infection [36,37,38]. In the study by Brackemeier et al., among 79 patients one developed miliary tuberculosis [39]. In the first study by Bertrand et al., one patient (0.9%) developed a mycobacterium tuberculosis infection, and one developed a mycobacterium paratuberculosis infection [11]. In the second study with the pooled cohort, no other patients developed tuberculosis [40].

Viana et al. reported on tuberculosis infection after kidney transplantation amongst 11,453 KT procedures within 16 years. Regarding maintenance immunosuppression 52% were receiving CNI/azathioprine (AZA); 34% received CNI/MPA, 7% received CNI/mTORi, and 0.3% belatacept/MPA (*n* = 34) and other combinations (*n* = 656). Among this cohort, 152 were diagnosed with tuberculosis. The incidence of TB was 0.94% with CNI/AZA, increasing to 1.60% with CNI/MPA (hazard ratio [HR] = 1.62), 2.85% with CNI/mTORi (HR = 2.45), and 14.7% with belatacept/MPA (HR = 13.1). Five belatacept-treated patients developed tuberculosis, of whom one died [74].

#### 6.2.3. Expert Opinion

Tuberculosis-related mortality is high among KTx. Although local epidemiology is an important factor, diagnostic/therapeutic challenges and immunosuppressive therapy may influence outcomes.

Experimental studies suggest that lymphocyte co-stimulation is necessary for granuloma formation [75]. It is unknown whether the belatacept-induced co-stimulation blockade is associated with reduced granuloma formation. Viana et al. reported on five belatacept-treated patients that developed TB in an endemic area (Brazil); four had lymph-node biopsies of which three showed granuloma formation with a concomitant positive bacilloscopy. Yet, two of these patients had disseminated TB, and one had extrapulmonary clinical presentations, suggesting compromised immune responses [74].

Before implementing belatacept-based therapy in populations at risk for tuberculosis, we recommend searching for latent tuberculosis by performing a Quantiferon test. If the test is positive, but without signs of active tuberculosis, we recommend treating with isoniazid for nine months before starting belatacept.

## 7. Belatacept Treatment in Association with Low-Dose CNIs and Infection Risk

In the BENEFIT and BENEFIT-EXT studies, although treatment with belatacept was associated with significantly better renal function and an improved metabolic profile, belatacept was also associated with higher rates and grades of early rejection [8,9]. Despite the observed benefits of this treatment, concerns about increased rates of rejection could limit the widespread adoption of belatacept as a primary therapy.

Adams et al. compared patients treated either with CNI-free, belatacept–based immunosuppression, or by a short course of tacrolimus (within the first five months post-transplantation) in combination with belatacept (Bela/Tacshort), or by a longer course of tacrolimus (up to nine months post-transplantation) combined with belatacept (Bela/Taclong) or tacrolimus/MPA. In that study, eGFR at four years was superior in belatacept-treated subjects compared to those that were receiving tacrolimus/MPA at post-transplantation, i.e., a mean difference of 15 mL/min. In terms of acute cellular-rejection rate, when a three-month course of tacrolimus was combined with belatacept (Bela/Tacshort), the rejection rate was significantly reduced, although not to the same level previously seen with standard tacrolimus-based therapy. Only when the tacrolimus taper was extended to nine months (Bela/Taclong) was rejection controlled to a level comparable to that seen with standard tacrolimus dosing.

In terms of safety, there were no significant differences in the rates of CMV or BKV viremias when comparing belatacept-based CNI-free and tacrolimus-based treatments. However, Adams et al. found a significantly higher rate of detectable CMV viremia in the belatacept-based CNI-free treatment compared to the other treatments, i.e., 27.8% vs. 14.2% (*p* = 0.02) This was probably due to the significantly higher rate of rejection and the subsequent administration of antithymocyte globulins with high doses of steroids to treat rejection episodes rather than a result of the initial immunosuppression regimen itself. BKV nephropathy was a rare event with no significant differences across the treatment groups. In addition, there were no differences with regard to serious infections that required hospitalization [76].

Recently, Gallo et al. assessed belatacept-based immunosuppression in association with low-tacrolimus dosing (trough levels ~2–3 ng/mL) in a specific population of KTx at high immunological risk and with a high medically complex profile (i.e., combined transplants). The rationale of this protocol was to combine the benefits of belatacept and reduced CNI exposure to minimize the risk of acute rejection. There were 19 patients; of these, 12 experienced viral (including CMV and EBV reactivation) or bacterial infections (incidence 0.062 episode/month of exposure considering a cumulative exposure time to belatacept of 257 months). Infection episodes were more common in early (94% of total events) versus late-converted KTx. One patient in the later conversion group experienced acute cholangitis secondary to biliary obstruction of the transplanted liver, with severe sepsis leading to graft failure. Hospitalization was needed in only 7/19 patients with significant clinical symptoms, and all recovered after appropriate therapy without belatacept interruption (except the one KTx with cerebral toxoplasmosis who stopped belatacept). *Pneumocystis* pneumoniae developed in one patient after the six months of prophylaxis was terminated [77].

### Expert Opinion

The study by Adams et al. shows that the adjunct of low-dose tacrolimus combined with belatacept, given in the first year post-transplantation, was associated with a huge drop in acute-rejection rate without increasing the risk of infection. More importantly, because of the higher rate of acute rejection in the belatacept-CNI free group, there were more cases of CMV-positive DNAemia in this group than in the tacrolimus group [57].

Gallo et al. showed that adding low-dose tacrolimus after a rescue conversion to belatacept in highly medically complex transplant patients was a feasible option and helped prevent acute rejection and improved graft function without substantially increasing infectious complications [77]. Even though the rate of infections was quite high in that study, this needs to be compared to when allograft failure leads to dialysis therapy, where there is a marked increased risk of mortality mostly due to infectious events [78,79]. This may be particularly true for transplant recipients with an organ other than a kidney (e.g., liver or pancreas) and for whom full immunosuppressive therapy has to be maintained.

## 8. Conclusions

Belatacept-treated patients respond poorly to SARS-CoV-2 vaccination. We still recommend three doses of mRNA-based vaccine. Hence, belatacept-treated KTx should maintain enhanced barrier measures. Vaccination of household members could also confer indirect protection to KTx. For those that do not develop anti-SARS-CoV-2 antibodies after three vaccine injections, we recommend casirivimab/imdevimab as prophylaxis, given by monthly injection.

Response to vaccination is, in general, a concern for belatacept-treated patients; further studies are thus needed to understand the underlying mechanisms that can explain the poor results observed.

Infected SARS-CoV-2 belatacept-treated patients tend to need more hospitalizations, both overall and in ICUs, and mortality is greater compared to CNI-treated patients. Regarding the available data, at present, with the COVID-19 infection, we do not recommend postponing belatacept injections or switching to another immunosuppressive regimen. Instead, we recommend organizing a dedicated infection-control protocol with stringent barrier precautions for patients that require regular outpatient infusions during the COVID-19 pandemic.

Although belatacept-based therapy has many clinical benefits, there are concerns regarding its safety in terms of acute cellular rejection and risk of infection. This systematic review suggests that even though there are issues that can affect the safety profile, it is still acceptable with regards to its benefits to avoid declining GFR and reduced graft survival, and/or an increased risk of cardiovascular disease. In addition, the studies that have raised concerns about the safety profile of using belatacept as a rescue treatment have been retrospective and uncontrolled. Hence, at present, the only way to use second-signal blockers in the setting of kidney transplantation is to use belatacept.

The main risk factors for developing an adverse event are early conversion from CNI to belatacept, a low GFR at conversion, older-recipient age, and steroid use. We recommend converting patients from CNI to belatacept at least six months after transplantation. Indeed, patients converted before six months have a significantly higher risk of developing an opportunistic infection (especially CMV disease and *Pneumocystis* pneumonia). The right timing to perform a conversion seems to be eight months post-transplantation.

If patients need to be converted within the first six months after transplantation, we recommend maintaining prophylaxis to prevent CMV and *Pneumocystis jiroveci* infections for three months in low-to-mild risk patients and for six months in high-risk patients.

Even though PTLD is still a concern for patients treated with belatacept, there is not an increased risk for PTLD in EBV-seropositive KTx as compared to CNI-treated KTx.

Thus, there is a need for prospectively controlled studies that can assess the risk of OIs when belatacept is given to KTx as rescue therapy.

## Figures and Tables

**Table 1:** Incidence of CMV disease.

					Belatacept De Novo		Belatacept Conversion	
Study (Reference)	Study Duration		Controlled Study	*n*=	CMV Disease/Infection	CMV Viremia	CMV Disease/Infection	CMV Viremia
BENEFIT [8]	12 months		Y					
		Bela MI		219	13 (6) +	NA		
		Bela LI		226	17 (8) +	NA		
		Cyclosporine		221	19 (9) +	NA		
BENEFIT-EXT [9]	12 months		Y					
		Bela MI		184	21 (11)	NA		
		Bela LI		175	24 (14)	NA		
		Cyclosporine		184	24 (13)	NA		
BENEFIT-EXT [13]	7 years		Y					
		Bela MI		184	2.20 *	NA		
		Bela LI		175	1.94 *	NA		
		Cyclosporine		184	1.71 *	NA		
Ferguson et al. [14]	1 year		Y					
		Bela + MMF		33	1 (3) +	NA		
		Bela + SRL		26	1 (3.8) +	NA		
Karadkhele et al. [24]	2 years		Y					
		Bela		104	NC	HR: 50 [49.5–50.5]		
		Tac		64	NC	HR: 34.4 [33.7–35.1]		
		Tac-MMF		30	2 (3.7) +	NA		
Rostaing et al. [15]	12 months		Y					
		Bela		84			2 (2) +	NA
		CNI		89			2 (2) +	NA
Grinyo et al. [16]	3 years		Y					
		Bela		84			0.84 *	NA
		CNI		89			0.85 *	NA
Darres et al. [17]	21.2 (0.1–337.1) months$		N	219			7 (2.7) +	NA
Gupta et al. [19]	2 years		N	56			6 (11.3) +	NA
Bertrand et al. [11]	31.9 ± 45.2 months$		N	280			18 (6.4) +	NA
Bertrand, Terrec et al. [21]	19 (0.13–133) months$		N	453			22 (4.8) +	74 (16.9)

+ Number of patients and %; * Incidence rates per 100 person years of study-drug exposure; $ Median time. Abbreviations: Bela, belatacept; MI, more intensive; LI, less intensive; Tac, tacrolimus; MMF, mycophenolate mofetil; SRL, sirolimus; CNI, calcineurin inhibitor; Y, yes; N, no; HR, hazard ratio; NA, not applicable; CMV, cytomegalovirus.

**Table 2:** Incidence of Pneumocystis jirovecii pneumonia.

Study (Reference)	Study Duration		Controlled Study	*n*=	Belatacept De Novo	Belatacept Conversion
BENEFIT [8]	12 months		Y			
		Bela MI		219	NA	
		Bela LI		226	NA	
		Cyclosporine		221	NA	
BENEFIT-EXT [9]	12 months		Y			
		Bela MI		184	1 (0.5) +	
		Bela LI		175	1 (0.5) +	
		Cyclosporine		184	0 (0) +	
BENEFIT-EXT [13]	7 years		Y			
		Bela MI		184	NA	
		Bela LI		175	NA	
		Cyclosporine		184	NA	
Ferguson et al. [14]	1 year		Y			
		Bela + MMF		33	NA	
		Bela + SRL		26	NA	
		Tac-MMF		30	NA	
Rostaing et al. [15]	12 months		Y			
		Bela		84		NA
		CNI		89		NA
Grinyo et al. [16]	3 years		Y			
		Bela		84		NA
		CNI		89		NA
Darres et al. [17]	21.2 (0.1–337.1) months$		N	219		3 (1.4) +
Gupta et al. [19]	2 years		N	56		NA
Bertrand et al. [11]	31.9 ± 45.2 months$		N	280		12 (4.3) +
Bertrand, Terrec et al. [21]	19 (0.13–133) months$		N	453		13 (2.8) +

+ Number of patients and %; * Incidence rates per 100 person years of study-drug exposure; $ Median time. Abbreviations: Bela, belatacept; MI, more intensive; LI, less intensive; Tac, tacrolimus; MMF, mycophenolate mofetil; SRL, sirolimus; CNI, calcineurin inhibitor; Y, yes; N, no; HR, hazard ratio; NA, not applicable.

**Table 3:** Overall risks for serious, fungal, viral, and bacterial infections.

Study (Reference)	Study Duration		Controlled Study	*n*=	Belatacept De Novo Overall Serious Infection	Belatacept De Novo Overall Fungal Risk	Belatacept De Novo Overall Viral Risk	Belatacept Conversion Overall Bacterial Risk	Belatacept Conversion Overall Fungal Risk	Belatacept Conversion Overall Viral Risk
BENEFIT [8]	12 months		Y							
		Bela MI		219	NA	NA	NA			
		Bela LI		226	NA	NA	NA			
		Cyclosporine		221	NA	NA	NA			
BENEFIT [12]	7 years		Y							
		Bela MI		74	10.36 *	7.89 *	17.53 *			
		Bela LI		71	6.71 *	4.23 *	16.89 *			
		Cyclosporine		73	3.14 *	2.54 *	3.01 *			
BENEFIT-EXT [9]	12 months		Y							
		Bela MI		184	NA	NA	NA			
		Bela LI		175	NA	NA	NA			
		Cyclosporine		184	NA	NA	NA			
BENEFIT-EXT [13]	7 years		Y							
		Bela MI		184	22.67 *	9.79 *	20.98 *			
		Bela LI		175	16.52 *	6.93 *	17.45 *			
		Cyclosporine		184	20.32 *	11 *	19.05 *			
Ferguson et al. [14]	1 year		Y							
		Bela + MMF		33	7 (21) +	5 (15) +	4 (12) +			
		Bela + SRL		26	4 (15) +	1 (4) +	2 (8) +			
		Tac-MMF		30	5 (17) +	2 (7) +	6 (20) +			
Rostaing et al. [15]	12 months		Y							
		Bela		84				20 (24) +	11 (13) +	11 (13) +
		CNI		89				17 (19) +	3 (3) +	12 (14) +
Grinyo et al. [16]	3 years		Y							
		Bela		84				10.21 *	9.73 *	14.60 *
		CNI		89				9.31 *	2.58 *	11.00 *
Darres et al. [17]	21.2 (0.1–337.1) months$		N	219				NA	80 (36.5) +	8 (3.7) +
Bertrand et al. [11]	31.9 ± 45.2 months$		N	280				9.8 *	NA	NA
Bertrand, Terrec et al. [21]	19 (0.13–133) months$		N	453				6.5 *	NA	NA

+ Number of patients and %; * Incidence rates per 100 person years of study-drug exposure; $ Median time. Abbreviations: Bela, belatacept; MI, more intensive; LI, less intensive; Tac, tacrolimus; MMF, mycophenolate mofetil; SRL, sirolimus; CNI, calcineurin inhibitor; Y, yes; N, no; HR, hazard ratio; NA, not applicable.

## Data Availability

Not applicable.

## References

[1] Starzl T.E., Fung J., Jordan M., Shapiro R., Tzakis A., McCauley J., Johnson J., Iwaki Y., Jain A., Alessiani M. (1990). Kidney transplantation under FK 506. JAMA.

[2] Myers B.D., Ross J., Newton L., Luetscher J., Perlroth M. (1984). Cyclosporine-associated chronic nephropathy. N. Engl. J. Med..

[3] Rodrigo E., Fernández-Fresnedo G., Valero R., Ruiz J.C., Pinera C., Palomar R., Gonzalez-Cotorruelo J., Gomez-Alamillo C., Arias M. (2006). New-onset diabetes after kidney transplantation: Risk factors. J. Am. Soc. Nephrol..

[4] Hošková L., Málek I., Kopkan L., Kautzner J. (2017). Pathophysiological mechanisms of calcineurin inhibitorinduced nephrotoxicity and arterial hypertension. Physiol. Res..

[5] Wojciechowski D., Vincenti F. (2016). Current status of costimulatory blockade in renal transplantation. Curr. Opin. Nephrol. Hypertens..

[6] Rostaing L., Malvezzi P. (2016). Costimulation Blockade in Kidney Transplantation: Beyond Belatacept. Transplantation.

[7] Malvezzi P., Jouve T., Rostaing L. (2016). Costimulation Blockade in Kidney Transplantation: An Update. Transplantation.

[8] Vincenti F., Charpentier B., Vanrenterghem Y., Rostaing L., Bresnahan B., Darji P., Massari P., Mondragon-Ramirez G.A., Agarwal M., Di Russo G. (2010). A phase III study of belatacept-based immunosuppression regimens versus cyclosporine in renal transplant recipients (BENEFIT study). Am. J. Transpl..

[9] Durrbach A., Pestana J.M., Pearson T., Vincenti F., Garcia V.D., Campistol J., Rial Mdel C., Florman S., Block A., Di Russo G. (2010). A phase III study of belatacept versus cyclosporine in kidney transplants from extended criteria donors (BENEFIT-EXT study). Am. J. Transpl..

[10] Terrec F., Jouve T., Naciri-Bennani H., Benhamou P.Y., Malvezzi P., Janbon B., Giovannini D., Rostaing L., Noble J. (2019). Late Conversion from Calcineurin Inhibitors to Belatacept in Kidney-Transplant Recipients Has a Significant Beneficial Impact on Glycemic Parameters. Transplant. Direct.

[11] Bertrand D., Chavarot N., Gatault P., Garrouste C., Bouvier N., Grall-Jezequel A., Jaureguy M., Caillard S., Lemoine M., Colosio C. (2020). Opportunistic infections after conversion to belatacept in kidney transplantation. Nephrol. Dial. Transplant..

[12] Boyarsky B.J., Werbel W.A., Avery R.K., Tobian A.A.R., Massie A.B., Segev D.L., Garonzi-Wan J.M. (2021). Antibody Response to 2-Dose SARS-CoV-2 mRNA Vaccine Series in Solid Organ Transplant Recipients. JAMA.

[13] Rozen-Zvi B., Yahav D., Agur T., Zingerman B., Ben-Zvi H., Atamna A., Tau N., Mashraki T., Nesher E., Rahamimov R. (2021). Antibody response to SARS-CoV-2 mRNA vaccine among kidney transplant recipients: A prospective cohort study. Clin. Microbiol. Infect..

[14] Cucchiari D., Egri N., Bodro M., Herrera S., Del Risco-Zevallos J., Casals-Urquiza J., Cofan F., Moreno A., Rovira J., Banon-Maneus E. (2021). Cellular and humoral response after mRNA-1273 SARS-CoV-2 vaccine in kidney transplant recipients. Am. J. Transplant..

[15] Grupper A., Rabinowich L., Schwartz D., Schwartz I.F., Ben-Yehoyada M., Shashar M., Katchman E., Halperin T., Turner D., Goykhman Y. (2021). Reduced humoral response to mRNA SARS-CoV-2 BNT162b2 vaccine in kidney transplant recipients without prior exposure to the virus. Am. J. Transplant..

[16] Benotmane I., Gautier-Vargas G., Cognard N., Olagne J., Heibel F., Braun-Parvez L., Martzloff J., Perrin P., Moulin B., Fafi-Kremer S. (2021). Low immunization rates among kidney transplant recipients who received 2 doses of the mRNA-1273 SARS-CoV-2 vaccine. Kidney Int..

[17] Chavarot N., Ouedrani A., Marion O., Leruez-Ville M., Vilain E., Baaziz M., Del Bello A., Burger C., Sberro-Soussan R., Martinez F. (2021). Poor Anti-SARS-CoV-2 Humoral and T-cell Responses After 2 Injections of mRNA Vaccine in Kidney Transplant Recipients Treated with Belatacept. Transplantation.

[18] Chavarot N., Morel A., Leruez-Ville M., Villain E., Divard G., Burger C., Serris A., Sberro-Soussan R., Martinez F., Amrouche L. (2021). Weak antibody response to 3 doses of mRNA vaccine in kidney transplant recipients treated with belatacept. Am. J. Transplant..

[19] Bertrand D., Hamzaoui M., Lemée V., Lamulle J., Hanoy M., Laurent C., Lebourg L., Etienne I., Lemoine M., Le Roy F. (2021). Antibody and T Cell Response to SARS-CoV-2 Messenger RNA BNT162b2 Vaccine in Kidney Transplant Recipients and Hemodialysis Patients. J. Am. Soc. Nephrol..

[20] Noble J., Langello A., Bouchut W., Lupo J., Lombardo D., Rostaing L. (2021). Immune Response Post-SARS-CoV-2 mRNA Vaccination in Kidney-Transplant Recipients Receiving Belatacept. Transplantation.

[21] Leibler C., Thiolat A., Hénique C., Samson C., Pilon C., Tamagne M., Pirenne F., Vingert B., Cohen J.L., Grimbert P. (2018). Control of humoral response in renal transplantation by belatacept depends on a direct effect on B cells and impaired T follicular helper-B cell crosstalk. J. Am. Soc. Nephrol..

[22] Chen J., Yin H., Xu J., Wang Q., Edelblum K.L., Sciammas R., Chong A.S. (2013). Reversing endogenous alloreactive B cell GC responses with anti-CD154 or CTLA-4Ig: Endogenous alloreactive B cell responses. Am. J. Transplant..

[23] Sattler A., Angermair S., Stockmann H., Moira Heim K., Khadzhynov D., Treskatsch S., Halleck F., Kreis M.E., Kotsch K. (2020). SARS–CoV-2–specific T cell responses and correlations with COVID-19 patient predisposition. J. Clin. Investig..

[24] O’Brien M.P., Forleo-Neto E., Musser B.J., Isa F., Chan K.C., Sarkar N., Bar K.J., Barnabas R.V., Barouch D.H., Cohen M.S. (2021). COVID-19 Phase 3 Prevention Trial Team. Subcutaneous REGEN-COV Antibody Combination to Prevent COVID-19. N. Engl. J. Med..

[25] Sidebottom D.B., Gill D. (2021). Ronapreve for prophylaxis and treatment of COVID-19. BMJ.

[26] Marx D., Moulin B., Fafi-Kremer S., Benotmane I., Gautier G., Perrin P., Caillard S. (2020). First case of COVID-19 in a kidney transplant recipient treated with belatacept. Am. J. Transplant..

[27] Kamar N., Esposito L., Hebral A.L., Guitard J., Del Bello A. (2020). Specific organization for in-hospital belatacept infusion to avoid nosocomial transmission during the SARS-CoV-2 pandemic. Am. J. Transplant..

[28] Elias M., Pievani D., Randoux C., Louis K., Denis B., Delion A., Le Goff O., Antoine C., Greze C., Pillebout E. (2020). COVID-19 Infection in Kidney Transplant Recipients: Disease Incidence and Clinical Outcomes. J. Am. Soc. Nephrol..

[29] Lau S.K.P., Lau C.C.Y., Chan K.-H., Li C.P.Y., Chen H., Jin D.Y., Chan J.F.W., Woo P.C.Y., Yuen K.Y. (2013). Delayed induction of proinflammatory cytokines and suppression of innate antiviral response by the novel Middle East respiratory syndrome coronavirus: Implications for pathogenesis and treatment. J. Gen. Virol..

[30] Huang C., Wang Y., Li X., Ren L., Zhao J., Hu Y., Zhang L., Fan G., Xu J., Gu X. (2020). Clinical features of patients infected with 2019 novel coronavirus in Wuhan. China. Lancet.

[31] Vincenti F., Lionel Rostaing M.D., Joseph Grinyo M.D., Kim Rice M.D., Steven Steinberg M.D., Luis Gaite M.D., Marie-Christine Moal M.D., Guillermo A., Mondragon-Ramirez M.D., Jatin Kothari M.D. (2016). Belatacept and Long-Term Outcomes in Kidney Transplantation. N. Engl. J. Med..

[32] Durrbach A., Pestana J.M., Florman S., Del Carmen Rial M., Rostaing L., Kuypers D., Matas A., Wekerle T., Polinsky M., Meier-Kriesche H.U. (2016). Long-Term Outcomes in Belatacept-Versus Cyclosporine-Treated Recipients of Extended Criteria Donor Kidneys: Final Results from BENEFIT-EXT, a Phase III Randomized Study. Am. J. Transplant..

[33] Ferguson R., Grinyó J., Vincenti F., Kaufman D.B., Woodle E.S., Marder B.A., Citterio F., Marks W.H., Agarwal M., Wu D. (2011). Immunosuppression with belatacept-based, corticosteroid-avoiding regimens in de novo kidney transplant recipients. Am. J. Transplant..

[34] Rostaing L., Massari P., Garcia V.D., Mancilla-Urrea E., Nainan G., del Carmen Rial M., Steinberg S., Vincenti F., Shi R., Di Russo G. (2011). Switching from calcineurin inhibitor-based regimens to a belatacept-based regimen in renal transplant recipients: A randomized phase II study. Clin. J. Am. Soc. Nephrol..

[35] Grinyó J.M., Del Carmen Rial M., Alberu J., Steinberg S.M., Manfro R.C., Nainan G., Vincenti F., Jones-Burton C., Kamar N. (2017). Safety and Efficacy Outcomes 3 Years After Switching to Belatacept from a Calcineurin Inhibitor in Kidney Transplant Recipients: Results from a Phase 2 Randomized Trial. Am. J. Kidney Dis..

[36] Darres A., Ulloa C., Brakemeier S., Garrouste C., Bestard O., Del Bello A., Sberro Soussan R., Dürr M., Budde K., Legendre C. (2018). Conversion to Belatacept in Maintenance Kidney Transplant Patients: A Retrospective Multicenter European Study. Transplantation.

[37] Yazdi M., Kahwaji J.M., Meguerditchian S., Lee R. (2021). Belatacept Conversion Protocols and Outcomes in Kidney Transplant Recipients. Transplant. Proc..

[38] Gupta G., Raynaud M., Kumar D., Sanghi P., Chang J., Kimball P., Kang L., Levy M., Sharma A., Bhati C.S. (2020). Impact of belatacept conversion on kidney transplant function, histology, and gene expression-A single-center study. Transpl. Int..

[39] Brakemeier S., Kannenkeril D., Dürr M., Braun T., Bachmann F., Schmidt D., Wiesener M., Budde K. (2016). Experience with belatacept rescue therapy in kidney transplant recipients. Transpl. Int..

[40] Bertrand D., Terrec F., Etienne I., Chavarot N., Sberro R., Gatault P., Garrouste C., Bouvier N., Grall-Jezequel A., Jaureguy M. (2020). Opportunistic Infections and Efficacy Following Conversion to Belatacept-Based Therapy after Kidney Transplantation: A French Multicenter Cohort. J. Clin. Med..

[41] Cippà P.E., Schiesser M., Ekberg H., van Gelder T., Mueller N.J., Cao C.A., Fehr T., Bernasconi C. (2015). Risk Stratification for Rejection and Infection after Kidney. Clin. J. Am. Soc. Nephrol..

[42] Bassil N., Rostaing L., Mengelle C., Kallab S., Esposito L., Guitard J., Cardeau-Desangles I., Weclawiak H., Izopet J., Kamar N. (2014). Prospective monitoring of cytomegalovirus, Epstein-Barr virus, BK virus, and JC virus infections on belatacept therapy after a kidney transplant. Exp. Clin. Transplant..

[43] Karadkhele G., Hogan J., Magua W., Zhang W., Badell I.R., Mehta A., Lyon M., Pastan S., Pearson T.C., Larsen C.P. (2021). CMV high-risk status and posttransplant outcomes in kidney transplant recipients treated with belatacept. Am. J. Transplant..

[44] Chavarot N., Divard G., Scemla A., Amrouche L., Aubert O., Leruez-Ville M., Timsit M.O., Tinel C., Zuber J., Legendre C. (2021). Increased incidence and unusual presentations of CMV disease in kidney transplant recipients after conversion to belatacept. Am. J. Transplant..

[45] Kotton C.N., Kumar D., Caliendo A.M., Huprikar S., Chou S., Danziger-Isakov L., Humar A. (2018). The third international consensus guidelines on the management of cytomegalovirus in solid-organ transplantation. Transplantation.

[46] Leeaphorn N., Garg N., Thamcharoen N., Khankin E.V., Cardarelli F., Pavlakis M. (2019). Cytomegalovirus mismatch still negatively affects patient and graft survival in the era of routine prophylactic and preemptive therapy: A paired kidney analysis. Am. J. Transplant..

[47] Xu H., Perez S.D., Cheeseman J., Mehta A.K., Kirk A.D. (2014). The allo- and viral-specific immunosuppressive effect of belatacept, but not tacrolimus, attenuates with progressive T cell maturation. Am. J. Transplant..

[48] Egli A., Kumar D., Broscheit C., O’Shea D., Humar A. (2013). Comparison of the effect of standard and novel immunosuppressive drugs on CMV-specific T-cell cytokine profiling. Transplantation.

[49] Basse G., Mengelle C., Kamar N., Guitard J., Ribes D., Rostaing L. (2007). Prospective evaluation of BK virus DNAemia in renal transplant patients and their transplant outcome. Transplant. Proc..

[50] Hirsch H.H., Knowles W., Dickenmann M., Passweg J., Klimkait T., Mihatsch M.J., Steiger J. (2002). Prospective study of polyomavirus type BK replication and nephropathy in renal-transplant recipients. N. Engl. J. Med..

[51] Hirsch H.H., Babel N., Comoli P., Friman V., Ginevri F., Jardine A., Lautenschlager I., Legendre C., Midtvedt K., Muñoz P. (2014). ESCMID Study Group of Infection in Compromised Hosts. European perspective on human polyomavirus infection, DNAemia and disease in solid organ transplantation. Clin. Microbiol. Infect..

[52] Saad E.R., Bresnahan B.A., Cohen E.P., Lu N., Orentas R.J., Vasudev B., Hariharan S. (2008). Successful treatment of BKV-viremia using reduction in immunosuppression without antiviral therapy. Transplantation.

[53] Schaub S., Hirsch H.H., Dickenmann M., Steiger J., Mihatsch M.J., Hopfer H., Mayr M. (2010). Reducing immunosuppression preserves allograft function in presumptive and definitive polyomavirus-associated nephropathy. Am. J. Transplant..

[54] Bischof N., Hirsch H.H., Wehmeier C., Steiger J., Mihatsch M.J., Hopfer H., Mayr M. (2019). Reducing calcineurin inhibitor first for treating BK polyomavirus DNAemia after kidney transplantation: Long-term outcomes. Nephrol. Dial. Transplant..

[55] Hirsch H.H., Yakhontova K., Lu M., Manzetti J. (2016). BK Polyomavirus Replication in Renal Tubular Epithelial Cells Is Inhibited by Sirolimus, but Activated by Tacrolimus Through a Pathway Involving FKBP-12. Am. J. Transplant..

[56] Benotmane I., Solis M., Velay A., Cognard N., Olagne J., Vargas G.G., Perrin P., Marx D., Soulier E., Gallais F. (2021). Intravenous immunoglobulin as a preventive strategy against BK virus viremia and BKV-associated nephropathy in kidney transplant recipients–Results from a proof-of-concept study. Am. J. Transplant..

[57] Trofe-Clark J., Sawinski D. (2016). BK and other polyomaviruses in kidney transplantation. Semin. Nephrol..

[58] Taylor A.L., Marcus R., Bradley J.A. (2005). Post-transplant lymphoproliferative disorders (PTLD) after solid organ transplantation. Crit. Rev. Oncol. Hematol..

[59] Opelz G., Döhler B. (2004). Lymphomas after solid organ transplantation: A collaborative transplant study report. Am. J. Transplant..

[60] Yost S.E., Ranga K.V., Kaplan B. (2011). Belatacept in renal transplantation. Dial. Transplant..

[61] Blazquez-Navarro A., Dang-Heine C., Wittenbrink N., Bauer C., Wolk K., Sabat R., Westhoff T.H., Sawitzki B., Reinke P., Thomusch O. (2018). BKV, CMV, and EBV Interactions and their Effect on Graft Function One Year Post-Renal Transplantation: Results from a Large Multi-Centre Study. EBioMedicine.

[62] Martin S.T., Powell J.T., Patel M., Tsapepas D. (2013). Risk of posttransplant lymphoproliferative disorder associated with use of belatacept. Am. J. Health Syst. Pharm..

[63] Santeusanio A., Bhansali A., De Boccardo G., Sehgal V., Delaney V., Florman S., Shapiro R. (2020). Conversion to belatacept maintenance immunosuppression in HIV-positive kidney transplant recipients. Clin. Transplant..

[64] El Sakhawi K., Melica G., Scemla A., Bertrand D., Garrouste C., Malvezzi P., Rémy P., Moktefi A., Ingels A., Champy C. (2020). Belatacept-based immunosuppressive regimen in HIV-positive kidney transplant recipients. Clin. Kidney J..

[65] Cambier M.L., Canestri A., Lependeven C., Peltier J., Mesnard L., Dahan K. (2019). Hepatitis B virus reactivation during belatacept treatment after kidney transplantation. Transpl. Infect. Dis..

[66] Iriart X., Belval T.C., Fillaux J., Esposito L., Lavergne R.A., Cardeau-Desangles I., Roques O., Del Bello A., Cointault O., Lagayssière L. (2015). Risk factors of Pneumocystis pneumonia in solid organ recipients in the era of the common use of posttransplantation prophylaxis. Am. J. Transplant..

[67] de Boer M.G., de Fijter J.W., Kroon F.P. (2011). Outbreaks and clustering of Pneumocystis pneumonia in kidney transplant recipients: A systematic review. Med. Mycol..

[68] de Boer M.G., Kroon F.P., le Cessie S., de Fijter J.W., van Dissel J.T. (2011). Risk factors for Pneumocystis jirovecii pneumonia in kidney transplant recipients and appraisal of strategies for selective use of chemoprophylaxis. Transpl. Infect. Dis..

[69] Phipps L.M., Chen S.C., Kable K., Halliday C.L., Firacative C., Meyer W., Wong G., Nankivell B.J. (2011). Nosocomial Pneumocystis jirovecii pneumonia: Lessons from a cluster in kidney transplant recipients. Transplantation.

[70] Struijk G.H., Gijsen A.F., Yong S.L., Zwinderman A.H., Geerlings S.E., Lettinga K.D., van Donselaar-van der Pant K.A.M.I., ten Berge I.J.M., Bemelma F.J. (2011). Risk of Pneumocystis jiroveci pneumonia in patients long after renal transplantation. Nephrol. Dial. Transplant..

[71] Jiang X., Mei X., Feng D., Wang X. (2015). Prophylaxis and treatment of Pneumocystis jiroveci pneumonia in lymphoma patients subjected to rituximab-contained therapy: A systemic review and meta-analysis. PLoS ONE.

[72] Brakemeier S., Dürr M., Bachmann F., Schmidt D., Gaedeke J., Budde K. (2016). Risk Evaluation and Outcome of Pneumocystis jirovecii Pneumonia in Kidney Transplant Patients. Transplant. Proc..

[73] Grinyó J., Charpentier B., Pestana J.M., Vanrenterghe Y., Vincenti F., Reyes-Acevedo R., Apanovitch A.M., Gujrathi S., Agarwal M., Thomas D. (2010). An Integrated Safety Profile Analysis of Belatacept in Kidney Transplant Recipients. Transplantation.

[74] Viana L.A., Cristelli M.P., Santos D.W., Tavares M.G., Dantas M.T.C., Felipe C.R., Silva H.T., Pestana J.M. (2019). Influence of epidemiology, immunosuppressive regimens, clinical presentation, and treatment on kidney transplant outcomes of patients diagnosed with tuberculosis: A retrospective cohort analysis. Am. J. Transplant..

[75] Teixeira H.C., Abramo C., Munk M.E. (2007). Immunological diagnosis of tuberculosis: Problems and strategies for success. J. Bras. Pneumol..

[76] Adams A.B., Goldstein J., Garrett C., Zhang R., Patzer R.E., Newell K.A., Turgeon N.A., Chami A.S., Guasch A., Kirk A.D. (2017). Belatacept Combined with Transient Calcineurin Inhibitor Therapy Prevents Rejection and Promotes Improved Long-Term Renal Allograft Function. Am. J. Transplant..

[77] Gallo E., Abbasciano I., Mingozzi S., Lavacca A., Presta R., Bruno S., Deambrosis I., Barreca A., Romagnoli R., Mella A. (2020). Prevention of acute rejection after rescue with Belatacept by association of low-dose Tacrolimus maintenance in medically complex kidney transplant recipients with early or late graft dysfunction. PLoS ONE.

[78] Li P.K.T., Chow K.M. (2012). Infectious complications in dialysis-epidemiology and outcomes. Nat. Rev. Nephrol..

[79] Tonelli M., Wiebe N., Knoll G., Bello A., Browne S., Jadhav D., Klarenbach S., Gill J. (2011). Systematic review: Kidney transplantation compared with dialysis in clinically relevant outcomes. Am. J. Transplant..

